# Identification of Coevolving Residues and Coevolution Potentials Emphasizing Structure, Bond Formation and Catalytic Coordination in Protein Evolution

**DOI:** 10.1371/journal.pone.0004762

**Published:** 2009-03-10

**Authors:** Daniel Y. Little, Lu Chen

**Affiliations:** 1 Department of Molecular and Cell Biology, University of California, Berkeley, California, United States of America; 2 Helen Wills Neuroscience Institute, University of California, Berkeley, California, United States of America; Michigan State University, United States of America

## Abstract

The structure and function of a protein is dependent on coordinated interactions between its residues. The selective pressures associated with a mutation at one site should therefore depend on the amino acid identity of interacting sites. Mutual information has previously been applied to multiple sequence alignments as a means of detecting coevolutionary interactions. Here, we introduce a refinement of the mutual information method that: 1) removes a significant, non-coevolutionary bias and 2) accounts for heteroscedasticity. Using a large, non-overlapping database of protein alignments, we demonstrate that predicted coevolving residue-pairs tend to lie in close physical proximity. We introduce coevolution potentials as a novel measure of the propensity for the 20 amino acids to pair amongst predicted coevolutionary interactions. Ionic, hydrogen, and disulfide bond-forming pairs exhibited the highest potentials. Finally, we demonstrate that pairs of catalytic residues have a significantly increased likelihood to be identified as coevolving. These correlations to distinct protein features verify the accuracy of our algorithm and are consistent with a model of coevolution in which selective pressures towards preserving residue interactions act to shape the mutational landscape of a protein by restricting the set of admissible neutral mutations.

## Introduction

A complete understanding of protein evolution will require full characterization of the many factors that determine the selective forces acting on each amino acid of a protein. Although it has long been hypothesized that the residues within a protein interact and influence each other's evolution, models of protein evolution, for simplicity and lack of sufficient data, have traditionally assumed that residues evolve independently of each other. However, the increasing power of bioinformatics and the increasing availability of genomic data offer a new opportunity to search for specific signals of coevolution.

The covarion (concomitantly variable codon) hypothesis, put forth by Fitch and Markowitz [1], postulated that, at any point during the evolution of a protein, only a small fraction of its residues are free to vary. As the freely varying sites mutate, however, interacting sites can switch between variable and invariant states. While Fitch and Markowitz emphasized this binary switching, they acknowledged that more subtle changes in selective pressures might occur. For example, in response to a mutation at a neighboring site, a residue might switch from varying among one set of amino acids to varying among another set. To encompass this broader conceptualization of coevolution, the covarion hypothesis can be restated as: at any point during the evolution of protein, only a small fraction of possible mutations are admissible, but as one site changes, it can alter the selective forces associated with other sites, thus altering the set of mutations that are selectively admissible at those site. This form of coevolutionary interaction could be recognized within a protein as residue pairs in which the variability at one site is dependent upon the amino acid state of the other.

Mutual information (MI) is a statistical measure of the codependency between two random variables. By considering the final amino acid states of a protein's residues, after a span of evolution, as discrete random variables, MI becomes a natural method for detecting codependencies between them. Using multiple sequence alignments (MSAs) to estimate the amino acid distribution at each site, MI quantifies how much uncertaintiy in the amino acid state at one site can be removed by knowledge of the amino acid state at another site.

The application of MI to sequence alignments was first introduced by Korber *et al.* as a means of identifying covarying sites in a viral peptide [2]. This approach was later extended to general proteins as a measure of coevolution [3]. Without refinement, however, MI yielded limited success and several attempts have been made to improve the measure [4]–[8]. Wollenberg and Atchely, for example, used parametric bootstrap simulations to model the effect of phylogenetic relationships on MI in the absence of coevolution [4]. Their approach, however, could not separate this global phylogenetic influence from the specific coevolutionary signal between a pair of sites [4]. Tillier and Lui attempted to capture biases acting on each site of a protein through an analysis of the total amount of interdependencies each site had across all other sites [5]. They, however, did not characterize the correlation between MI and their measure of this bias. Their method of removing this bias from MI may, therefore, have been suboptimal and may have hindered the accuracy of their algorithm. These and the other researchers have emphasized the need to quantify and effectively remove the poorly understood biases that are hindering the efficacy of MI as a measure of coevolution [4]–[9].

Since the “true” coevolutionary history of a protein cannot be experimentally determined, measures of coevolution cannot currently be directly tested. This complicates the validation of any measure and necessitates the use of indirect evidence. A correlation between predicted coevolving residue-pairs and protein structure is the most common evidence offered to support the accuracy of an algorithm [2], [4]–[8], [10]–[17]. Indeed, many researchers who develop algorithms for quantifying covariability between sites abandon coevolution as their primary goal and instead focus on the algorithm's potential as a tool for structure prediction, in particular contact prediction [12], [13], [18], [19]. Still, the correlation that these algorithms yield with protein structure is likely mediated by their capacity to accurately measure coevolution combined with an inherent tendency for physically close residues to interact evolutionarily.

Demonstrating that a measure's predicted coevolving residues are further correlated to additional relevant protein features aside from structure can, by an argument of parsimony, greatly increase the support for that measure as it limits the range of potential non-coevolutionary explanations. Towards this end, researchers occasionally offer examples of coevolving residues that they consider to be functionally relevant or near functionally relevant sites [8], [14]–[16]. Such correlations should, however, be evaluated carefully and with consideration of two factors. First, site-specific biases, such as conservation, may artificially conflate the coevolutionary measure of functionally relevant residues. Second, the appropriate controls are rarely given to demonstrate that the highlighted examples represent a true trend. Once a correlation is shown to be statistically significant and not the result of artifactual biases, it not only supports the accuracy of a measure but also provides insight into the nature of coevolution.

In this article, we offer a refinement of MI as a measure of coevolution that removes a strong non-coevolutionary influence and accounts for differences in within-site variability. We demonstrate a high correlation between our predicted coevovling residues and protein structure, which even extends to quaternary structures. We also demonstrate a significant trend for those residues that are annotated as participating directly in a protein's catalytic activity to coevolve with each other. Going beyond these two more commonly considered correlations, we offer a novel measure of the propensity for each pair of the 20 amino acids to be found at coevolving sites, which we term their “coevolution potentials”. We found that amino acid pairs known to interact in bond formation exhibited the strongest coevolution potentials, providing a unique correlation for our measure with the known biochemistry of proteins that had not previously been explored. We concluded by demonstrating directly that our measure surpasses previous methods in its degree of structural correlation, a standard comparison for evaluating measures of coevolution [6], [11], [20].

## Results

### Refining mutual information as a measure of coevolution

To develop a statistical framework for measuring coevolution, we began by modeling the propensity for each amino acid to evolve at a site in a protein as a discrete random variable with 20 possible outcomes representing the 20 amino acids. To look for interdependencies between two sites (i.e. two random variables), we then considered their joint distributions. If the propensity for a particular amino acid to evolve at one site is completely independent from the amino acid state of the other site, then the joint distribution will simply be a product of the two single distributions, and the entropy (a statistical measure of disorder) of the joint distribution will equal the sum of the entropies for the two single distributions. If, however, the propensity for a particular amino acid to evolve at one site is completely determined by the amino acid state at the other site, then the two single distributions and their joint distribution will all be equivalent with equal entropies. MI is a statistical quantity that measures the codependency of two random variables by examining how much less entropy (i.e. more order) there is in their joint distribution than would be expected if the two distributions were completely independent.

**Figure 1:**
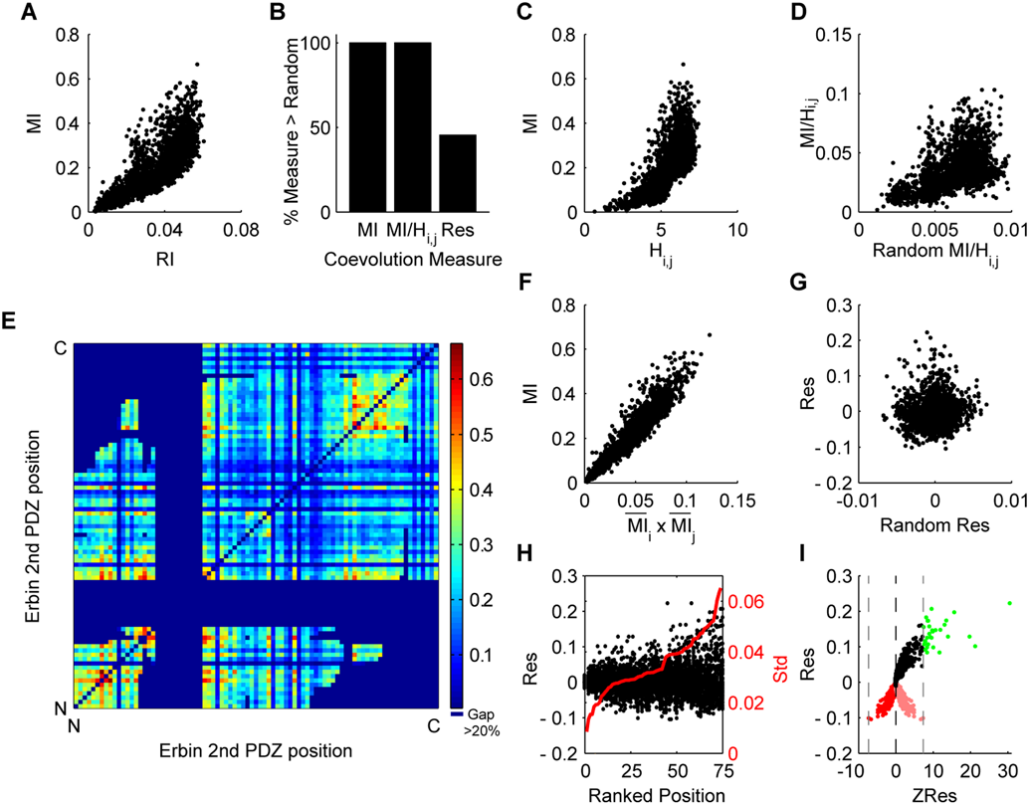
Measuring coevolution without bias. (A) MI scores are correlated to random information scores (RI) in which all coevolutionary and phylogenetic relationships have been removed by random perturbations (RI is an average over 300 randomizations). This demonstrates that MI suffers from a non-phylogenetic bias. (B) The percentage of tested residue pairs that have coevolution measures higher than their average random measure (Standard deviations over 300 randomizations are plotted but are too small to be visualized). Phylogenetic biases induce high MI and MI/H_i,j_ scorings, which are unobtainable from randomized results. (C) MI is correlated to H_i,j_. (D) MI/H_i,j_ is correlated to its randomized values (same MI/H_i,j_ measure but with all coevolutionary and phylogenetic relationships removed from sites by random perturbation of amino acids). MI/H_i,j_ is therefore subject to non-phylogenetic biases. (E) A colorimetric representation of MI scores between pairs of residues in the 2^nd^ PDZ domain of the Human Erbin protein. The striated appearance highlights a large variation in basal MI values between sites. Residue positions are aligned from the N-terminus to the C-terminus. Red = high MI, Blue = low MI, Darkest Blue = untested (>20% gaps). (F) MI is correlated to 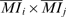. (G) Res is not correlated with its randomized values. (H) Positions are ranked in order of increasing variance in Res scores (red line indicates deviation of Res scores) and the distribution of Res scores are plotted. Higher variation at a site increases the likelihood of false indentification of coevolution at that site [5]. (I) ZRes scores are calculated as the product of the z-scores of a Res value relative to its distribution across each site. Light red points represent residue pairs where both z-scores were negative. The ZRes score for such sites are taken as the negative of the product of the z-scores (dark red points). The negative of the lower bound of ZRes (gray lines) is a cutoff for choosing coevolving residues (green points).

In order to calculate a reliable numerical estimate for MI, many instances of the random variables are necessary (i.e. many copies of a protein evolving independently but under the same selective pressures). We approximated this by considering the sequences of an MSA as instances of our random variable. The sequences of an MSA, however, fail to meet the assumption of independent evolution. While the stabilization of a mutation in an ancestral protein represents only one evolutionary event, it would be considered, under MI-analysis, as representing an independent event for each descendant protein of that ancestor in the MSA. This treatment of a single event as multiple independent events should act as a source of bias that increases the mutual information among residues. By independently mixing the amino acids at each site among the sequences of an MSA, we can calculate random mutual information (RI) scores in which all coevolutionary signals and potential phylogenetic biases have been removed. As an example, we plotted the MI scores for each pair of amino acid sites in the Pfam full alignment of 5612 PDZ domains against their average RI scores from 300 randomizations (Figure 1A; Pfam ID: PF00595 [21]). The RI score for two sites was almost never higher than their MI score (Figure 1B; less than 1 residue pair out of all 2193 pairs per randomization). Since we would expect most residues to have strong evolutionary interactions with only a limited number of sites [6], the increased mutual information scores of the unperturbed MSA relative to the randomized MSA likely represent the influence of phylogenetic relationships.

Surprisingly, we also noticed that MI is significantly correlated to RI (R = 0.7892) despite having removed the coevolutionary and phylogenetic interactions. This suggests that MI is further subject to additional, non-phylogenetic biases, which we term the stochastic bias.

Gloor *et al.* and Martin *et al.* noted that MI is highly correlated to joint entropy (H_i,j_; R = 0.7323, Figure 1C), and thus chose to normalize their measure by dividing MI by H_i,j_ [8], [9]. However, when we applied the same randomizing method to this derived measure, we found that the normalized measure and its randomization were still highly correlated (R = 0.5669, Figure 1D). Normalizing MI by H_i,j_ thus failed to completely remove the stochastic biases. Furthermore, the tendency for a measured MI/H_i,j_ value to be higher than the randomized measure was equivalent to that of the MI values (Figure 1B; this equivalency is a mathematical consequence), suggesting that the phylogenetic biases were still to some degree present. MI/H_i,j_ is therefore an inefficient normalization method, and a variable with greater explanatory power over the biases would be preferred.

A colorimetric representation of the MI scores for the PDZ alignment (relative to the Human Erbin 2^nd^ PDZ domain whose structure has been solved) exhibited a striated appearance, indicative of dramatically varying general levels of MI at different sites (Figure 1E). We captured the basal MI level for a site by averaging the MI scores for all residue pairings with that site (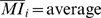 MI across row *i* in Figure 1E). Large differences in the 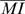 of different sites are unlikely to represent true coevolutionary patterns since most sites should only coevolve strongly with a limited set of partner sites and the basal coevolutionary interactions between sites should be similar [6]. 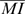 is therefore likely to capture site-specific biases. Such biases could potentially arise from the positioning within the phylogenetic tree of the mutations at a particular site. For example, a site that that mutates just after a branching point that evenly bifurcates the tree (and thus yields a more even distribution of the two alleles) is likely to have higher 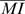 than a site that mutates at a more distal branch point where the allelic distributions would be more skewed. Other uncharacterized stochastic biases may also be contained in 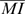. We used the product of the 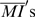 at two sites (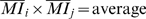 MI across row *i* x average MI across column *j* in figure 1E) to capture the combined bias for that pair of sites. In order to evaluate the influence of the combined biases on the MI scores, we plotted MI against 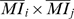 for all pair of sites (Figure 1F). We discovered that the two quantities were highly correlated in a strong linear relationship (R = 0.9477), with 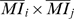 explaining 90% of the variation in MI. This correlation persisted even when the higher 50% of MI scores across a site were removed from the calculation of 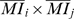 (R = 0.9301), demonstrating that the correlation was not a result of high measurements for the top “truly” coevolving sites. Thus 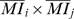 is a non-coevolutionary variable with high explanatory value towards MI, and therefore likely contains the biases that mask the true coevolutionary signal. To remove the influence of 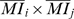 from MI, we used a least-squares regression and calculated the residual (Res) of MI over 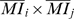. The Res measure did not correlate with randomized results (Figure 1G; R = 0.0863), suggesting that we had successfully removed the stochastic bias. Furthermore, about 50% of all residue pairs exhibited random scores higher than the measured Res values (Figure 1B). These results suggest that Res represents a measure of coevolution in which biases associated with MI (i.e. phylogenetic and stochastic) have been removed. Note that this quantification of the biases was purely empirical.

We noticed that the variation in the residuals of the linear regression of MI over 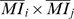 displayed heteroscedasticity: increased variation with increasing MI (Figure 1F). To examine how differences in variation might be influencing our Res scores, we plotted the distribution of Res scores for each site, sorting the sites by increasing variance (Figure 1H). While average Res values tended to be similar across all sites, the variation at each site differed dramatically. A plot of the standard deviation in Res scores for each site against the entropy of that site revealed that the two are correlated, suggesting that sites with more variation in amino acid composition (i.e. more entropy) have an increased tendency to vary in their Res value (R = 0.4516, p<0.0001, Figure S1). Without correction, more variable sites would have a wider distribution of Res values and thus an increased chance to randomly surpass selection threshold. To adjust for these differences in variation, for each pair of sites, *i* and *j*, we compared their Res score to the distribution of Res scores across site *i* as well as the distribution of Res scores across site *j*. We then calculated the z-scores (number of standard deviations above or below the mean) for the Res score relative to each of these two distributions. Finally, in order to account simultaneously for the relative position of the Res score in both distributions, we defined a new measure, ZRes, as the product of these two z-scores (analogous to the Pearson correlation). Thus ZRes is a normalized measure of the position of the Res score for a pair of sites relative to the distributions of Res scores across each of those sites. ZRes is large in magnitude when the Res value for a pair of sites are at the ends of both distributions and small when it is close to the middle of each distribution. If a Res value sits at the low end of both distributions, it would indicate low coevolutionary interactions. The associated z-scores, however, would both be negative making their product positive (Figure 1I, light-red). To distinguish such low coevolution pairs from those that lie at the positive ends (high coevolution) of both distributions, we reversed the signs of their ZRes score (Figure 1I, dark red). Since these residue pairs represent the distribution of ZRes scores for non-coevolving residues, their maximum value in magnitude (ZLB, the lower bound of ZRes; Figure 1I, left gray line) offered us a useful selection threshold (-ZLB; Figure 1I, right gray line) for choosing coevolving sites with signals above background variation (Figure 1I, green).

### Coevolution in PDZ domains

**Figure 2:**
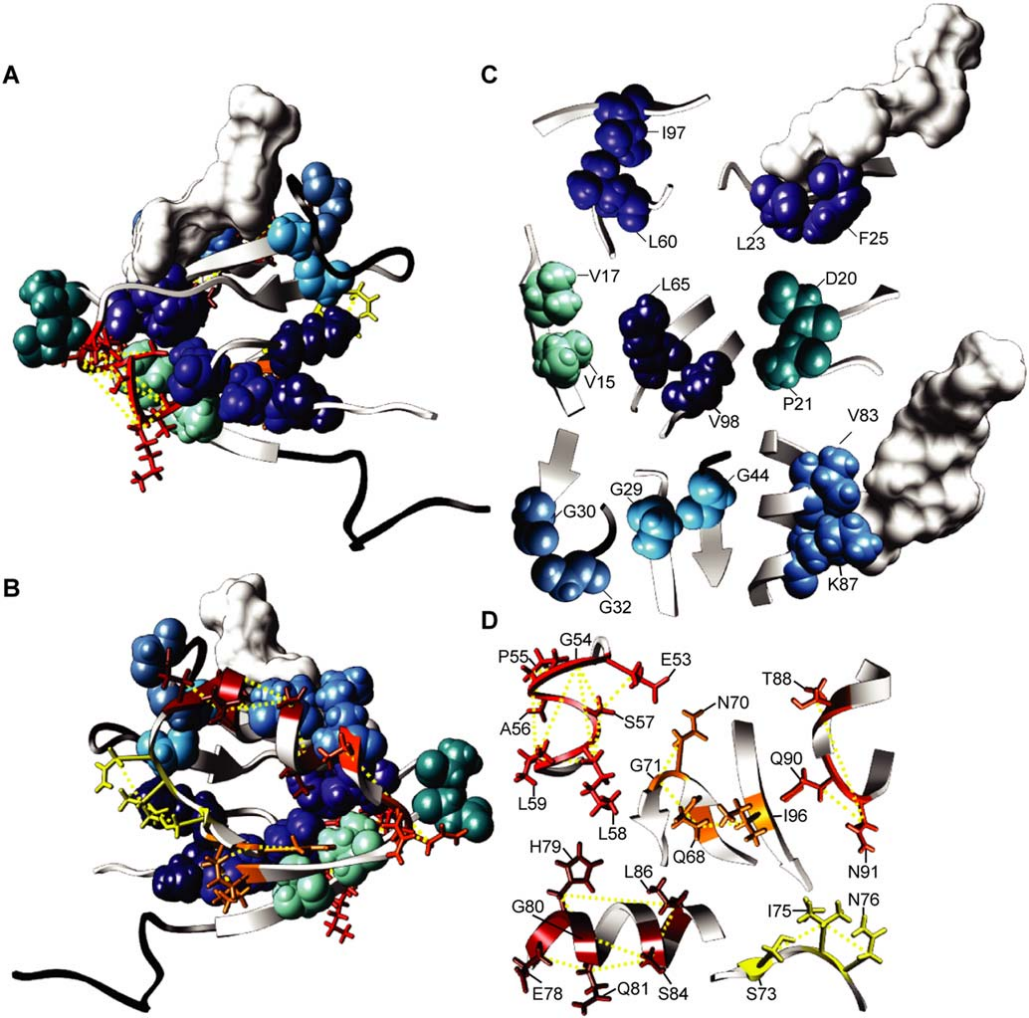
Coevolving residues in the 2^nd^ PDZ domain of Human Erbin. (A) The structure of 2^nd^ PDZ domain of Human Erbin with peptide ligand. Coevolving networks of at least 3 residues are depicted as balls-and-sticks in shades of red with dashed yellow lines connecting the coevolving pairs. Isolated pairs of coevolving residues are depicted as spheres in shades of blue. The molecular surface of the peptide ligand is depicted in white. Black ribbons represent untested residues (>20% gaps). (B) Backside of A. (C) Isolated pairs of coevolving residues. (D) Networks of 3 or more coevolving residues.

The PDZ domain is commonly found in scaffolding proteins where it serves as a binding site for specific peptide sequences in target proteins. 80–90 amino acids in length, its small size makes it amenable towards easily visualizing the coevolutionary pairs identified by our algorithm. The structure of the 2^nd^ PDZ domain of the Human Erbin protein has been solved and shown to be similar in general topology to other PDZ representatives [22] (PDB ID: 1N7T [23], [24]). To examine how the coevolving pairs of residues identified by our algorithm might be interacting within the structure of the PDZ domains, we mapped all residue pairs with ZRes scores higher than the -ZLB cutoff onto the structure of the Erbin 2^nd^ PDZ domain (Figures 2A & 2B; visualizations done with UCSF Chimera [25]). Isolated pairs of residues that were identified as only coevolving with each other are depicted as space-filled spheres, each pair a different shade of blue (Figure 2C). Networks of three or more residues connected by coevolutionary interactions are depicted in ball-and-stick form with dashed yellow lines connecting the β carbons of the coevolving pairs (Figure 2D). In total we identified 30 coevolving pairs falling into 13 networks and involving 39 unique residues, nearly half of the tested residues.

**Figure 3:**
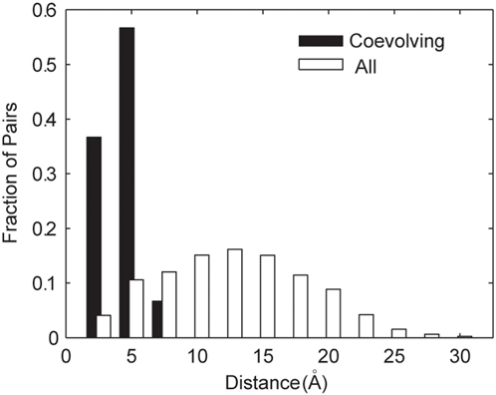
Distribution of distances between coevolving residues of PDZ domains. The fraction of coevolving (black bars) or all (white bars) residue pairs that lie within the specified interval of physical distance from each other is depicted.

The close physical proximity between each coevolving residue pair is quite striking. We plotted the distribution of distances between pairs of coevolving residues and compared it to the distribution of distances between all tested pairs of residues (Figure 3). We found that the interacting residues were significantly closer together (median distances: 2.88 Å (coevolving), 11.30 Å (all); p<1×10^−16^, 2-sample Kolmogorov-Smirnov (K-S test)). We interpret this as arising from a tendency for coevolving residues to be close to each other combined with the ability of our ZRes measure to accurately detect signals of coevolution. Interestingly, while many of the coevolving residues where found to lie in the same secondary structure (e.g. Val-83 and Lys-87 which align on one side of the only α-helix; Figure 2C), several examples were also found of residues interacting between secondary structures (e.g. Gln-68 and Ile-96 interacting between the 4^th^ and 6^th^ β-sheets; Figure 2D).

### Coevolution in 1592 Pfam families

The Pfam website (http://pfam.sanger.ac.uk/) maintains a database of non-overlapping alignments of well-characterized protein families and domains [21]. In order to test the generality of our results from the PDZ alignment across a larger set of proteins, we downloaded 1592 Pfam full alignments chosen based on the criteria that they contained at least 500 sequences and at least two sites with less than 20% gaps.

**Figure 4:**
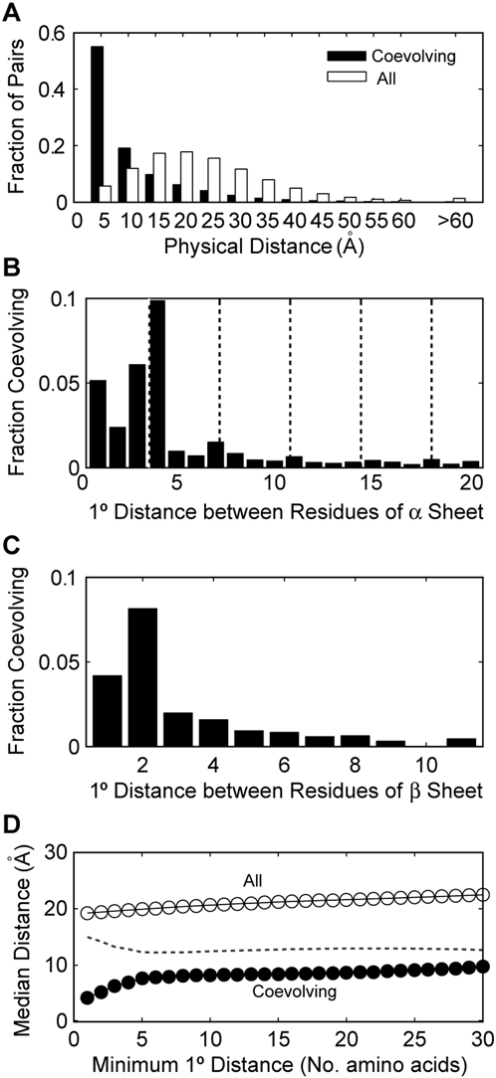
Coevolving residues correlate with structure. (A) The fraction of coevolving (black bars) or all (white bars) residue pairs that lie within the specified interval of physical distance from each other across 1592 Pfam families. (B) The fraction of residue pairs lying within the same α-helix and having the specified primary sequence separation that are coevolving. Neighboring residues have a primary (1°) distance of 1. Multiples of 3.6 have been superimposed onto the plot (dashed lines) to indicate typical spacing between turns of an α-helix. (C) The fraction of residue pairs lying within the same β-sheet and having the specified primary sequence separation that are coevolving. (D) The median distance of coevolving (closed circles) or all (open circles) residue pairs with the indicated minimum primary sequence separation. The dotted line depicts the difference between all and coevolving median distances.

Examining all 1592 alignments, the strong linear relationship between MI and 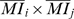 persisted across alignments (mean R = 0.9442±0.0340), showing that 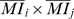 consistently explained much of the variability in MI. Utilizing our ZRes measure, we identified 126,085 coevolving residue pairs (out of 18,073,342) with scores above the -ZLB cutoff. While our coverage of the set of all tested residue pairs was low (0.7%), on average, 57.1%±19.6% of the tested residues for each protein family were identified as coevolving with at least one other residue. This suggests that our algorithm is selective on the pairings of residues and not biased towards specific single sites. To test whether the identified coevolving residues correlated with physical distance, we obtained structural data on representative members for 1240 of the 1592 Pfam alignments [23]. A single representative structure was chosen for each alignment. Figure 4A shows the distribution of the distances between the 86,084 identified coevolving pairs of residues present in the representative structures. For comparison, the distribution of distances between all 12,203,471 tested pairs of residues in the 1240 crystal structures is also shown. Indeed, the coevolving residues were significantly closer together (p<1×10^−307^, K-S test) with a median distance of 4.3 Å as compared to a median of 19.2 Å for all tested residue pairs. 56% of the identified coevolving residues were within 6 Å of each other, indicative of direct physical contact [8]. In comparison, only 7% of all tested residue pairs were in a similar range of contact. Furthermore, to test whether these results could have arisen from a bias in our measure towards selecting a specific set of sites that as a population tended to be close together, we examined the set of all sites identified as coevolving with at least one other site. The median distance between pairs of sites amongst this set (19.3 Å) was no different than the total distribution for all tested pairs of sites nor was the percentage of site pairs in contact (7%). This demonstrates that our correlation to physical structure is specifically dependent on the pairing of identified coevolving residues and not the result of single-site biases. We therefore interpret these results as emerging from the accuracy of our algorithm at identifying coevolving residues paired with the tendency for direct structural interactions to strongly influence residue coevolution.

To further explore correlations between coevolving residues and structural interactions, we next considered secondary structure. Of the 86,084 coevolving residue pairs, 14,653 (17.0%) were found to lie in a common α-helix or β-sheet. In comparison, only 3.8% of all residue pairs were identified as lying in a common α-helix or β-sheet, suggesting that residues interacting within a secondary structure have an increased tendency to influence each other's evolution. We noticed, from the PDZ domain, that coevolutionary interactions tended to be spaced as to align along the same side of the α-helix or β-sheets. To test the generality of this observation, we considered all coevolving pairs of residues where both residues lied in the same α-helix (Figure 4B) or the same β-sheet (Figure 4C) and determined their primary sequence separation. The results are given as a fraction of the total number of residue pairs that were located within a common secondary structure of the respective type (α-helix or β-sheet) and separated by the given primary distance. Residues within an α-helix exhibited a strong peak at 3 and 4 amino acids primary distance, coincident with the first turn of an alpha-helix (3.6 amino acids, first dashed line in Figure 4B). The propensity to coevolve quickly died off for primary distances past 4 amino acids, probably because subsequent helix turns become further and further away from each other in the molecular structure. Still a subtle peak can be seen every 3–4 amino acids consistent with the approximate 3.6 amino acids per turn characteristic of α-helices [26]. Even though the correlation for β-sheets was not as strong, it did exhibit a strong peak for residues that were separated by only a single amino acid (i.e. the closest residues to align on the same side of a β-sheet).

We next tested whether coevolving residues that were distant in primary sequence were still close in tertiary structure. To examine this possibility, we restricted our analysis to residue pairs separated by a minimal primary sequence distance and recalculated the median physical distance of predicted coevolving pairs (Figure 4D). Even at a minimum of 30 amino acids primary distance separation, coevolving sites were significantly closer in physical distance (median: 9.8 Å) than the total distribution of sites with that minimal separation (median: 22.5 Å; p<1×10^−307^, K-S test; Figure 4D). Similar statistical significance was obtained for all primary distance separations from 1 to 30 (p<1×10^−307^, separate K-S tests for each minimum primary distance). Example molecular distance distributions (for the 10 and 30 primary distance minimums) are given in the supplemental data (Figure S2). For increasing minimum primary distance thresholds from 1 through 6, a moderate decrease in the difference between the median coevolving distances and the median for all sites was observed (Figure 4D, dashed line). This is perhaps due to the significance of secondary structural relationships in this range of primary sequence separation. Past a minimum primary distance of 6, however, the differences between the coevolving sites and all sites become constant suggesting that the tendency towards coevolution is indifferent to the degree of primary sequence separation beyond those separations strongly correlated to interactions within a secondary structure.

We then examined the influence of sequence length and alignment size on the accuracy of our algorithm. We approximated the accuracy of our algorithm in identifying coevolving residues by its accuracy in contact prediction (the percentage of identified coevolving residue pairs separated by at most 6 Å). Across alignments, the total number of tested residue pairs that contacted each other scaled with the protein's effective sequence length (the square-root of the number of tested residue pairs; Figure S3A). This led to a strong correlation between the percentage of tested residue pairs that were in contact and the reciprocal of effective sequence length (R = 0.8428; Figure S3B). Thus, one might expect that the ability to preferentially identify those residue pairs in contact as coevolving over those not in contact would decrease with increases in effective sequence length. However, the robustness of our results led us to speculate that our use of the -ZLB selection threshold potentially adjusted for this bias. Indeed, the contact accuracy for identified coevolving residue pairs was much less correlated to the reciprocal of effective sequence length than were the percentages of all tested residue pairs contacting (R = 0.1976; Figure S3C), though there was still a slight overall gain in performance for shorter proteins. This suggests that our algorithm effectively compensated for the decreased representation of coevolving residue pairs (which should increase linearly with protein length) relative to the total number of tested residue pairs (which increased quadratically with protein length). Finally, we also found a subtle but significant positive correlation between the contact accuracy for identified coevolving residue pairs and the number of sequences in an alignment, suggesting that larger alignments yielded increased accuracy (R = 0.1003, p<0.001; Figure S3D). These correlations to contact prediction accuracy most likely reflect a corresponding correlation to coevolution prediction accuracy.

### Coevolution potentials

**Figure 5:**
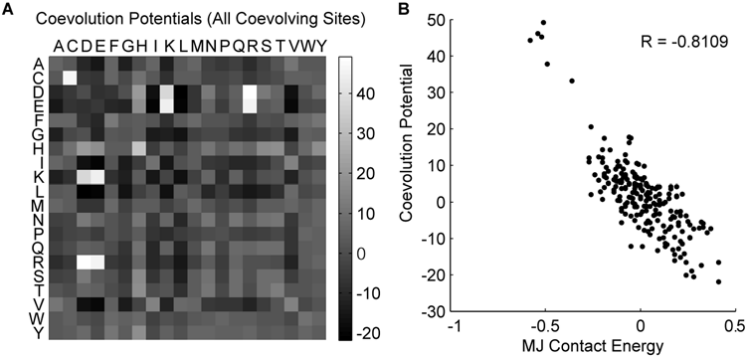
Coevolution potentials between the amino acids. (A) Coevolution potentials calculated using all identified coevolving sites. (B) Coevolution potentials are correlated with the MJ contact energies.

Having applied our algorithm to a large set of proteins, we next wanted to search for possible trends in the amino acid compositions of coevolving sites. We therefore developed a measure of the propensity for strongly coevolving sites to be composed of each of the 210 possible pairings of the 20 amino acids, which we termed the coevolution potentials between the amino acids. For each pair of coevolving sites (with ZRes≥-ZLB), we calculated the frequency of each amino acid pair amongst the sequences of the corresponding MSA. We then weighted these frequencies by the ZRes score between those sites. These weighted values were calculated for all coevolving pairs and then summed. To account for biases in the distribution resulting from differences in the frequency of occurrence of each amino acid in the alignments, we determined the statistically expected outcome for repeating this calculation using randomly selected residue pairs but weighting them by the ZRes values of the original coevolving pairs. Our final coevolution potentials represent the standard score for the coevolving amino acid pairs relative to their expected values and variance under the random process (Figure 5A; no randomizations were performed, expected value and variance were calculated mathematically).

The 11 highest coevolution potentials (in decreasing order) were found to be between: Asp-Arg, Cys-Cys, Glu-Arg, Glu-Lys, Asp-Lys, His-His, Asp-His, His-Thr, His-Tyr, His-Glu, His-Ser (Table S1). The high coevolution potentials of the acid-base amino acid pairs (Asp-Arg, Glu-Arg, Glu-Lys, Asp-Lys) suggest that coevolutionary forces may act to maintain balanced ionic charges or specific ionic interactions. Similarly, the series of pairings with histidine may be highlighting the importance of maintaining acceptor[A]-donor[D] interactions in side-chain hydrogen bonds (His[A/D]-His[A/D], Asp[A]-His[D], His[A/D]-Thr[A/D], His[A/D]-Tyr[A/D], His[D]-Glu[A], His[A/D]-Ser[A/D]) [27]. Interestingly, as noted, histidine along with serine, tyrosine, and threonine represent a class of amino acids whose side chains can act both as hydrogen donors and accepters [27]. We speculate that these amino acid pairs represent an evolutionary ‘pivot-point’ around which acceptors and donors can reverse roles. We also note that histidine is unique in its ability to act both as a acid and base at physiological pHs suggesting that it may represent a similar crux for the transitions in acid-base pairs. Finally, coevolutionary pressures selecting against the reactive thiol group of cysteine may explain the high coevolution potential of the Cys-Cys pair.

The known importance of ionic interactions, hydrogen bonds, and disulfide bonds in protein structure also offer a biochemical explanation for the correlation between physical structure and our coevolution scores. Indeed our coevolution potentials showed high correlation to Miyazawa and Jernigan's contact energies, which describe the potential for amino acid pairs to be in physical contact with each other (MJ; R = −0.8109, Figure 5B) [28]. It seemed possible that the high coevolution potentials of certain amino acid pairs were actually a result of their correlation to physical proximity rather than an explanation for it. To test this possibility, we recalculated the coevolutionary potentials but only considered those pairs of sites that were already known to be within 6 Å of each other in the representative structure. Since these contacting coevolution potentials were normalized to the expected results for randomly selected contacting site-pairs, they represent the tendency for each amino acid pair to be found at coevolving sites above and beyond the biases due to physical proximity. The results show that even once physical proximity has been removed as a bias in the potentials, acid-base, cysteine-cysteine, and hydrogen bond acceptor-donor pairs still dominate the coevolutionary interactions (Figure S4A; Table S1). Indeed the contacting coevolution potentials still strongly correlate with the MJ contact energies (R = −0.7394, Figure S4B). We interpret these results as suggesting that a common form of coevolution arises from selective pressures to maintain important bond-forming interactions, which are inherently short-ranged. Such selective pressure would help to explain the tendency for coevolving sites to be close to each other.

While the correlation between our coevolution potentials and the MJ contact energies is consistent with our findings that pairs of coevolving residues tend to be close together, there were still many pairs of coevolving residue that were distant from each other in the representative structures. To investigate the amino acid compositions of these distant coevolving sites, we again recalculated our coevolution potentials considering only those residue pairs that were greater than 6 Å apart in their representative structures (Figure S4C, Table S1). To our surprise, much of the correlation to bond-forming interactions (i.e. high coevolution potentials of acid-base pairs and of cysteine-cysteine) and to the MJ contact energies was preserved (R = −0.6601, Figure S4D). These results suggested that of those residue pairs identified as coevolving and distant in representative structures, some may nonetheless still be close in a different context such as different protein conformations, different representative structures, or contacts between copies of the protein in multi-protein complexes. We examine this last possibility in the following “Inter-molecular coevolution” section.

Our inability to fully separate distant coevolving residue pairs from those that interact at close-range makes it difficult to ascertain which amino acid pairs are more commonly found in long-range coevolutionary interactions. Nevertheless, the distant coevolution potentials did exhibit an increased ranking for pairs of aromatic amino acids in preference over several of the hydrogen-bond forming pairs identified by the earlier potentials: His-His (rank 6), Trp-Tyr (rank 7), Phe-Tyr (rank 8), and Trp-Trp (rank 10). It is unclear to us why these aromatic amino acid pairs were particularly represented among the distant coevolving residues.

### Inter-molecular coevolution

**Figure 6:**
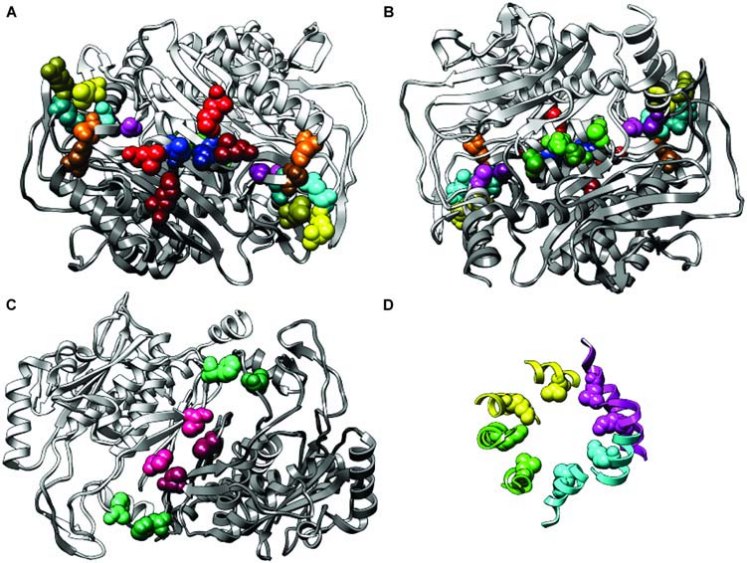
Inter-molecular interactions between coevolving residues of the chorismate synthase tetramer. (A–C) Coevolving residues are highlighted by the same hue. Light residues are from chain A. Dark residues are from chain D (panels A and B) or chain C (panel C). (B) The back side of the structure depicted in panel A. (D) A pair of coevolving residues forming a planar ring at the center of the tetramer. Each molecule of chorismate synthase is depicted in a different color.

A surprising result from an examination of the coevolving residues of chorismate synthase offered a partial explanation as to why coevolving residue pairs that are distant in their representative structures are still correlated to the MJ contact energies. Chorismate synthase is a homotetramerizing protein important in the synthesis of aromatic compounds in bacteria, and its crystal structure has been solved (PDB ID: 1UM0) [29]. Examining the distribution of distances between residues within a single chain of chorismate synthase (chain A in the representative crystal structure), we again found that the coevolving residue pairs were significantly closer together than all tested residue pairs (Figure S5; p<1×10^∧^−48, K-S test; median distances: 5.78 Å (coevolving), 23.63 Å (all)). Interestingly, when we began mapping the strongest coevolving sites onto the crystal structure of the chorismate synthase tetramer, we found that many of them were directly apposed to each other across the dimer interfaces (Figure 6A–C). Amongst the top 50 ZRes scoring residue pairs, 34 residue pairs (68%) were found to be contacting each other (≤6 Å apart) within a single molecule of chorismate synthase (chain A). Of the 16 pairs that were not in intra-molecular contact, 9 were found to be in contact between molecules of the tetramer (Figure 6A–C) and an additional pair was found to form a planar ring at the interface of the four chains (Lys-232 and Leu-349; Figure 6D). Many of these coevolving residues were predicted by UCSF Chimera to form inter-molecular hydrogen bonds (data not shown) [25]. Taken together with the previous results, this suggests that residues may coevolve to maintain structural interactions both within and between protein molecules.

**Figure 7:**
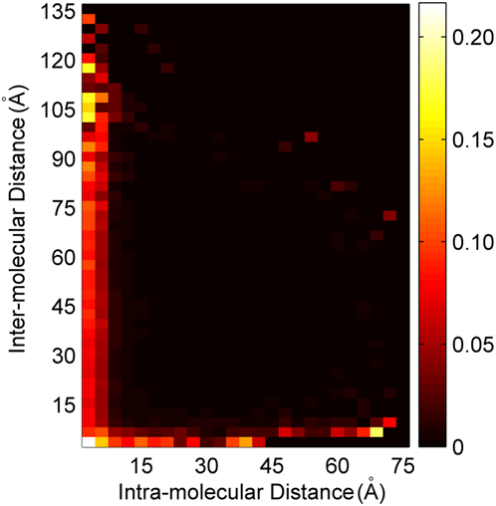
Joint distribution of intra-molecular and inter-molecular distances between coevolving residues. 532 protein and domain alignments whose representative PDB structures contained multiple copies of the corresponding peptide were used for the analysis. The color of each cell depicts the fraction of all residues pairs lying within the specified intervals of intra-molecular and inter-molecular distances that are coevolving. Coevolving pairs are particularly prevalent amongst residues pairs that lie in close physical proximity to each other either intra-molecularly or inter-molecularly.

To further test this hypothesis, we identified 532 alignments whose representative crystal structure contained multiple copies of the aligned protein. Since the formation of protein crystals inherently imposes a multimerization of the peptides, we restricted our analysis to only those chains in the structure identified as being part of a biologically relevant assembly (“REMARK 350” in PDB files) [23]. Plotting the joint histogram of intra-molecular and inter-molecular distances for the coevolving sites normalized to the joint histogram for all tested sites, we found that the coevolving site pairs were particularly represented amongst those that were physically close either within a protein or between interacting copies of the protein (Figure 7). Of all 9207 residues pairs that were within 6 Å of each other in inter-molecular distance, over 10% (1167 pairs) of them were identified as coevolving. In comparison, only 0.7% of all site-pairs (distant or close) were selected as coevolving. The percentage of intra-molecularly contacting residue pairs (less than 6 Å apart) rose from 6.23% for all tested pairs to 58.20% for coevolving pairs, while the percentage of inter-molecularly contacting residues rose from 0.34% to 2.59%. These results clearly demonstrate the importance of inter-molecular interactions in the coevolution of residues.

### Coevolution of Catalytic Sites

We next examined whether catalytic sites, being direct participants in the functional role of enzymatic proteins, exhibited specific coevolutionary tendencies. Two lines of evidence have commonly been offered to support the hypothesis that catalytic sites elicit or require strong coevolutionary interactions: 1) examples of catalytic sites coevolving with other (not necessarily catalytic) sites are highlighted, or 2) a prevalence of non-catalytic coevolving sites within 10 Å of a protein's active site is demonstrated [6], [8], [14]–[16]. Statistical support verifying that these trends surpass random expectations, however, is often not offered. Furthermore, care should be given towards considering what biases in an algorithm might inappropriately increase coevolutionary measures for catalytic sites. For example, since low entropy is correlated with high conservation, the normalization of MI by H_i,j_ discussed earlier might bias the measure towards selecting evolutionarily conserved sites [8].

The Catalytic Site Atlas (CSA) provides information on which residues in a PDB structure are implicated in the direct catalytic activity of an enzyme [30]. Of the 1240 representative crystal structures utilized in this study, a total of 645 catalytic sites in 257 proteins have been identified in the CSA. Using our measure, we found that of the catalytic sites, 61.6% (397) were identified as coevolving with at least one other (not necessarily catalytic) site, and 97.8% (631) were within 10 Å of an identified coevolving pair of sites. Control experiments, however, revealed that these results were not specific to the catalytic residues. Indeed, the 61.2% of all sites had coevolving partners and the 98.8% of all sites were within 10 Å of a coevolving pair. We therefore conclude, that while catalytic sites are indeed amongst the coevolving sites, they have no increased propensity to be coevolving in general.

**Figure 8:**
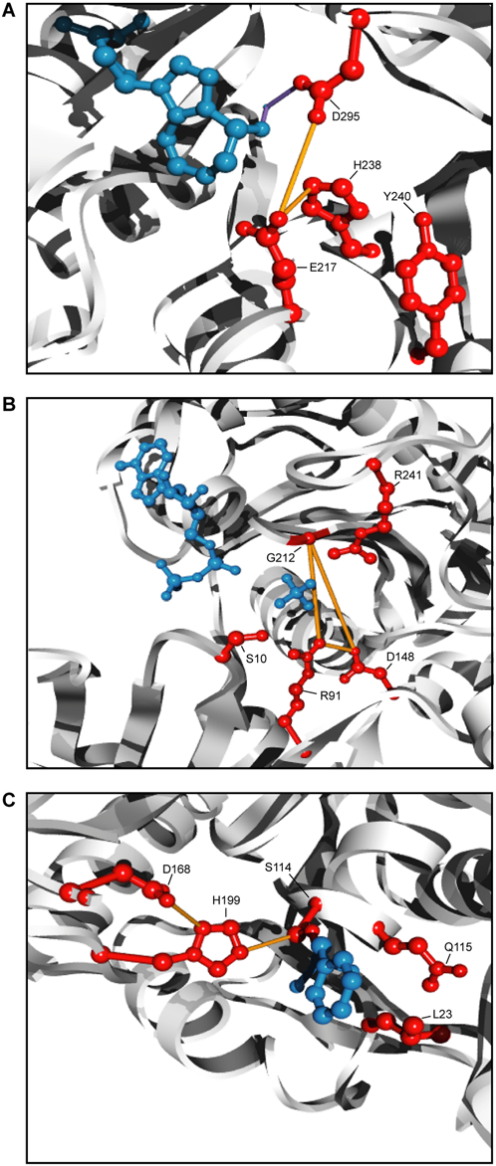
Coevolution between catalytic sites. All catalytic sites annotated by the CSA [30] and tested for coevolution (i.e.≤20% gaps) are depicted in red. The protein backbones are depicted as a white ribbon. Coevolving catalytic residues are connected by orange lines. (A) Active site of murine adenosine deaminase (PF00962, PDB 1a4l) [38]. The inhibitor, pentostatin, and a coordinating Zn^2+^ ion are depicted in blue. The coordinating interactions with Zn^2+^ is depicted as purple lines [38]. (B) The nucleotide binding site of *Methanosarcina thermophila* acetate kinase (PF00871, PDB 1g99) [39]. The bound ADP molecule and a sulfate ion are depicted in blue. (C) Active site of *Pseudomonas fluorescens* carboxylesterase (PF02230, PDB 1aur) [40]. The inhibitor, phenylmethylsulfonyl fluoride, is covalently bound to Ser114 and its phenylmethylsulfonyl moiety is depicted in blue.

While catalytic sites did not demonstrate any increased tendency towards having coevolutionary partners in general, we wondered whether catalytic sites tended to coevolve specifically with each other. Of the 257 PDB structures with CSA entries, 175 contained at least two catalytic sites and were used for our subsequent analysis. We found that 61 of these PDB structures contained at least one pair of catalytic sites identified as coevolving with each other. In total, there were 90 such coevolving pairs of catalytic sites, representing 11% of all possible catalytic site pairs (793). To determine whether this propensity for catalytic sites to coevolve with one another was significantly higher than random expectations, for each representative structure, we selected random sites equal in number to the number of catalytic sites and asked how many of these random sites coevolved with one another. Over 2000 randomizations, the average total number of coevolving pairs of random sites was only 6.5±2.7 (0.8%), significantly fewer than the number of coevolving pairs of catalytic sites identified (in all 2000 randomizations, the randomly selected sites never shared 90 or more coevolutionary pairing; given a normal fit of the random results log transformed to satisfy normalcy, we calculated the probability of getting 90 or more coevolving pairs to be less than 1×10^−16^). Repeating the analysis with random sites chosen under the requirement that all selected sites for a protein be contacting each other in the representative structure, only 56.7±7.2 (7.2%) were identified as coevolving, showing that the tendency for catalytic sites to coevolve could not be completely explained by any tendency to be located near each other at active sites (p<1×10^−16^). Three example proteins containing coevolving catalytic sites have been depicted in Figure 8.

### Comparison to Previous Methods

To compare the performance of our algorithm to previously published methods, we considered several measures that, like ours, attempt to detect residue coevolution by quantifying the covariability between sites. We had chosen to utilize an MI-based approach because MI is well established in Information Theory. Other methods for quantifying the covariability, however, have been adapted towards coevolution detection. The Observed Minus Expected Squared (OMES) approach developed by Kass and Horovitz utilized a χ^2^ goodness-of-fit test to identify site pairs at which the observed distribution of amino-acid pairs diverged significantly from expectation [10], [11]. The McLachlan Based Substitution Correlation (McBASC) approach developed by Göbel *et al.* looked for correlations in the degrees of divergence for paired substitutions at two sites [11]–[13]. Furthermore, a recent report from Dunn *et al.* independently developed a measure of coevolution (MIp) analogous to our Res measure [6]. A subtle difference lies in how 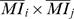 is removed from the MI score. Dunn *et al.* utilized an insightful mathematical proof, to estimate the relationship between MI and 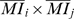. We, on the other hand, directly calculated the residuals of the linear regression of the measure on the bias. Dunn *et al.*, however, failed to account for the differences in within-site variability addressed by our ZRes measure [6].

**Figure 9:**
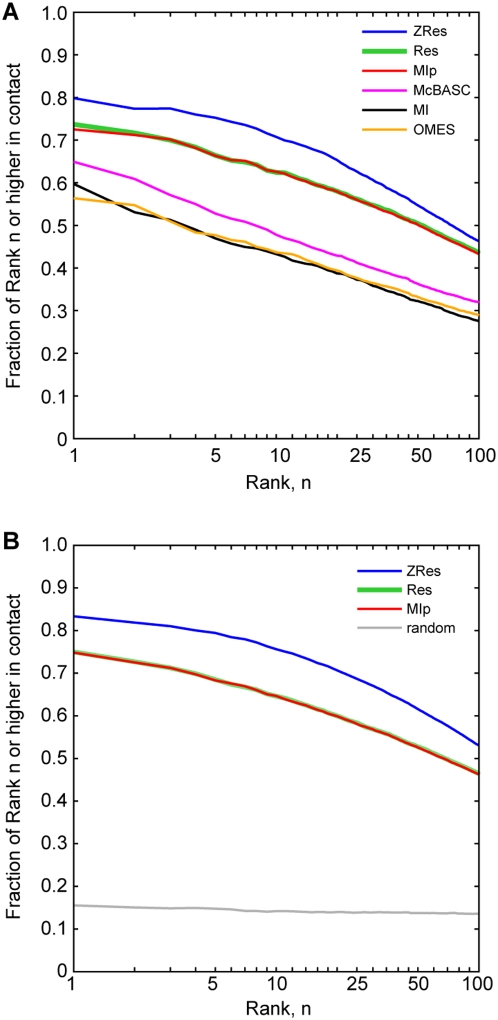
Comparison of ZRes to other measures of coevolution. (A) To ease processing load, calculations were limited to the 424 alignments with representative structures for which the product of the protein sequence length and alignment size was less than or equal to 100,000. Following the analysis performed previously [5], all residue pairs were ranked from highest to lowest ZRes score. For ranks 1 up to 100, the fraction of residue pairs at or higher than each rank lying within 6 Å of each other was calculated. The average of this contact accuracy across all alignments was then plotted (blue). The process was repeated with the Res (green), OMES (brown), McBASC (magenta), MIp (red), and MI (black) measures. (B) as in A, but utilizing all 1240 alignments with representative crystal structure. The results from one randomization of residue pair rankings are plotted in black. Statistical significance was assessed by Friedman's nonparametric 2-way ANOVA for measure effects on selectivity after factoring out rank effects. All pair-wise comparison in both A and B were significant except between MIp and Res.

To compare our algorithm to these previously developed methods, we used contact prediction accuracy as an approximate correlate of coevolution prediction accuracy. Since none of these algorithms utilize structural data (including primary sequence order) and since none of them are based on known signals for contact prediction, any correlation with structural data should arise from their ability to recognize coevolving sites combined with a tendency for coevolving sites to be close together (or for close residues to be coevolving). Contact prediction therefore is a reasonable approximation of algorithm accuracy. In order to make the comparisons, each measure was used to rank all tested site pairs for each analyzed protein family, and the percentage of the top ranking site pairs contacting in their representative structures were calculated. Our ZRes measure out-performed both OMES and McBASC (p<1×10^−16^, Friedman's nonparametric two-way ANOVA; Figure 9A). Furthermore, whereas MIp and Res performed equally well, they both under-performed ZRes, showing that our controls for heteroscedasticity significantly improved the measure (p<1×10^−16^, Friedman's nonparametric two-way ANOVA; Figures 9A and 9B). Since shorter protein sequences have a large fraction of residue pairs in contact with each other (Figure S3B), we repeated the analysis adjusting for sequence length by normalizing the number of top scoring site pairs chosen for each protein family by the length of the protein sequence (Figure S6). Again, ZRes performed significantly better than all other measures (p<0.05 for 1% protein sequence length down to p<1×10^−5^ for 32% protein sequence length, K-S test).

## Discussion

Since it is presently infeasible to directly test whether two residues in a protein have interacted evolutionarily, the accuracy of an algorithm for measuring coevolution can only be assessed through its correlation with independently determined features. Even when such correlations exist and are shown to be statistically significant, care must be taken in considering whether a non-coevolutionary variable or bias in the measure might underlie the correlation. Once such non-coevolutionary explanations have been ruled out, these correlations both offer validation for an algorithm, and provide insights into the nature of coevolution.

The coevolutionary interactions predicted by our algorithm show a strong correlation with physical structure, namely coevolving pairs of residues tend to be in close proximity to each other. Since our algorithm utilizes no information on structural data or even the primary sequence order, and since this correlation does not arise from a bias towards identifying a specific set of sites as having coevolving partners, this supports the accuracy of our algorithm and suggests that residues lying in close physical proximity are more likely to influence the selective pressure acting on each other. In addition, the high performance of our algorithm at predicting residue contacts may in the future offer a means of improving protein structure prediction algorithms. Indeed several methods for combining coevolutionary measures in structural predictions have been previously described and would be interesting to pursue in future studies [18], [19], [31].

Our calculation of coevolution potentials between the 20 amino acids offers new insights into the role of bond-forming interactions in evolution. The results suggest that bond forming residue pairs may commonly face particularly strong coevolutionary selective pressure towards maintaining these bonds. Although such selective pressure might suggest conservation, it is important to note that coevolution requires variation. Thus the capacity for similar bonds to be formed by different amino acid pairs may provide a means to maintain these necessary interactions while tolerating variation. The predominance among coevolving residues of acid-base pairs could also indicate a common coevolutionary selective pressure towards maintaining a balance of ionic charges. The high coevolution potential of the cysteine-cysteine pair may also suggest a common need to protect against the high reactivity of the cysteine thiol group.

Our coevolution potentials for “distant” residues highlight the importance of context in investigating algorithms for coevolution detection. While the coevolution of distant, oppositely charged residues might be explained as maintaining a global ionic balance, the persistence of the cysteine-cysteine pair among the highest coevolution potentials would be hard to explain if such residues were indeed distant. More likely they are only distant in one context but are close in another. The physical interaction of these residues may be revealed if we examine their structures in different contexts such as different representative proteins within an alignment or different conformational states of a protein. As one example (Figure 7), we have shown that the structural interactions between seemingly distant coevolving sites can be revealed upon consideration of inter-molecular distances within a protein complex. An interesting consideration for future directions would be to extend these results to protein-protein interaction predictions, potentially as a supplement to already existing algorithms [17], [32]–[34].

Coevolving residues are often expected to participate directly in the catalytic function of a protein. Researchers therefore often draw attention to those residues known to play important roles in a protein's catalytic activity that they identify as having coevolutionary partners. They, however, often fail to offer controls showing that random sites are not equally likely to be identified as coevolving, nor controls showing that their selection of catalytic sites does not result from single-site biases (such as a bias towards selecting conserved sites). For our measure, we have shown that catalytic sites, as determined by the CSA, do not have an increased propensity to coevolve in general. We, however, do reveal an increased tendency for these catalytic sites to coevolve with each other above random chance. That is, catalytic sites selectively coevolve more strongly with other catalytic sites. Since this correlation to coevolution was identified only for pairs of catalytic sites and was not present when considering catalytic sites one at a time, it is not likely to arise from site-specific biases. These findings underscore the importance of residue coordination in realizing and maintaining an optimal enzymatic activity.

To explain the competing roles of selective pressure and variation, both necessary for coevolution, we offer a coevolutionary extension of the Neutral Model of Evolution offered by Kimura [35], and King and Jukes [36]. We hypothesize that coevolutionary change predominantly occurs through the genetic drift of neutral mutations at interacting sites, but the set of neutral mutations available to those sites is largely restricted to maintain structural and catalytic interactions. When multiple means of retaining such interactions are available (e.g. multiple ways of forming similar bonds), these selective forces would not be so constraining that they prevent any variation at the sites. As nearly-neutral mutations stabilize, the interactions between each residue change, altering the set of subsequently available neutral mutations. Given that variability is important in the detection of coevolution, those residue pairs that most strongly cooperate in defining the shape of a protein's mutational landscape without severely restricting it will exhibit the strongest coevolutionary signal. This might further explain why catalytic sites do not exhibit a general increase in tendency to coevolve. Perhaps many catalytic sites are too constrained to allow any variation, and thus do not allow any covariation.

## Methods

### Multiple sequence alignments

The full alignments for the PDZ domain family (PF00595) and chorismate synthase family (PF01264) were downloaded from the Pfam database (http://pfam.sanger.ac.uk/, Pfam 21.0) [21]. 1592 PFAM additional full alignments were also downloaded and utilized in our large-scale analysis (Pfam 22.0). These full alignments were chosen based only on the criteria that they contained at least 500 sequences and at least one site with fewer than 20% gaps. Importantly, no residue of any sequence is represented in more than one PFAM alignment, protecting our large-scale analysis from redundancy [37]. Of these alignments, 1240 had solved structures (http://www.pdb.org/) [23], [24]. A single representative structure was chosen for each alignment without regard to the coevolution results. The complete list of Pfam IDs for the 1592 full alignments, PDB IDs for the 1240 representative structures, and the sequence number in the alignments of the representative members is available in the supporting material (Table S2).

### Calculating MI

Given a multiple sequence alignment (MSA), let p_i_ be the vector of length 20 whose entries are the frequencies of the 20 amino acids amongst all the sequences at position i ignoring gaps. We treat p_i_ as an estimator for the random variable representing the likelihood of each amino acid evolving at position i. Next we let p_i,j_ be a 20 by 20 matrix representing the joint distribution of each ordered amino acid pair at positions i and j. Entropy, *H*_i_, is a measure of the uncertainty associated with p_i_ and is given by:
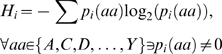
(1)H_i_ has a minimum value of 0, i.e. no uncertainty, when all sequences in the MSA have the same amino acid at position i. and it increases as the amino acid frequencies become more evenly distributed with a maximal value when all 20 amino acids are equally represented. The joint entropy, H_i,j_, between p_i_ and p_j_ is simply the entropy of the joint distribution p_i,j_ and is given by:
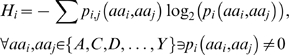
(2)If p_i_ and p_j_ are completely independent, then H_i,j_ = H_i_+H_j_. H_i,j_ decreases as p_i_ and p_j_ become more codependent and is a minimal when the amino acid at p_i_ completely determines what amino acid must occur at p_j_.

Finally, the mutual information of p_i_ and p_j_, MI_i,j_, is a statistical quantification of the interdependency between them and is given by:
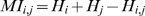
(3)Derived in this fashion, MI can be interpreted as the increase, due to codependency, in the certainty of the joint outcome over the expected certainty assuming complete independence.

It is important to note that gaps in the MSA can both skew the represented phylogenies at a site and decrease the sample size for estimating frequencies. For this reason, if any pair of sites had more than 20% of the sequences in the MSA gapped at either positions then no MI score, nor any of the derived measures of coevolution, were calculated for that pair. Such gapped pairs were thus left untested for any coevolutionary relationship.

### Derived coevolutionary measures

Following the logic laid out in the Results Section, to remove the biases associated with MI, we began by calculating the average MI for each position i:
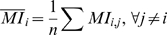
(4)Where n is the number of positions j for which an MI score was calculated between i and j (<20% gaps). We found a strong linear relationship between MI_i,j_ and 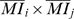 (Figure 1F). This correlation persisted even when MI_i,j_ or the top 5 MI values for each site were removed from the determination of 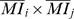 (data not shown). Since each site would be expected to coevolve with only a few other sites, their average MI with all sites would not be expected to contain coevolutionary signal. 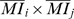 is therefore a confounding variable that potentially contains the biases, phylogenetic and stochastic, that affected MI. To remove the influence of this non-coevolutionary variable from MI, we calculated the linear least squares regression of MI_i,j_ against 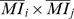 and took the residual of each MI_i,j_ over this line of best fit as a new measure of coevolution, Res_i,j_.

A second refinement was made to our measure to account for the higher variability in MI, and thus in Res, at some sites over others. We started by considering Res_i,j_ as a member of two larger sets of Res scores: the Res scores between i and all other sites, Res_i,*_, and the Res scores between j and all other sites, Res_*,j_. We next let Z_i_(j) be the z-score for Res_i,j_ relative to the population Res_i,*_:
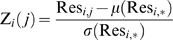
(5)Here, μ(Res_i,*_) and σ(Res_i,*_) represent the mean and standard deviation of Res_i,*_ respectively. An analogous z-score, Z_j_(i) was calculated for Res_i,j_ relative to the population Res_*,j_. To normalize for the variability at both i and j, we calculated a final measure:
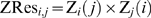
(6)If Res_i,j_ is less than the mean value for both populations, Res_i,*_ and Res_*,j_, both its z-scores would be negative. This was problematic since their multiplication would then become positive. To avoid this, we interpreted only position pairs where both Z_i_(j) and Z_j_(i) were positive as exhibiting a coevolutionary signal. At the same time, since the z-scores for a population distribute around zero, by letting ZLB (ZRes lower bound) be the most negative value obtained by ZRes, -ZLB was a natural cutoff threshold for our positive ZRes signals. More explicitly, to include the positional pairs where both Z_i_(j) and Z_j_(i) are negative, we calculate ZLB as:
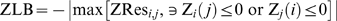
(7)


The MI/H_i,j_ and MIp measures of coevolution were calculated as described by Gloor *et al.* and Dunn *et al.* respectively [6], [8]. OMES and McBASC were calculated as described by Fodor and Aldrich [11].

### Randomized coevolution scores

For each pair of positions i and j, 300 randomized coevolution measures (for MI, MI/H_i,j_, and Res) were calculated by randomly mixing the amino acids across the sequences of the MSA that were not gapped at either.

### Structural distances

Intra-molecular distances between residues in a structure were calculated as the minimum distance between any pair of atomic coordinates from the two residues. Inter-molecular distances were calculated for all alignments whose representative sequence was present on multiple chains annotated by the PDB file as part of a biological unit (PDB REMARK 350) [24]. The inter-molecular distances were calculated as the minimum atomic distance between two residues across all pairs of chains (not including same chain distances).

### Coevolution potentials

For each pair of residues identified as significantly coevolving, we determined the frequency of the 210 unordered pairs of the 20 amino acids for those sites in the corresponding MSA. We then weighted these frequencies by the ZRes score for that pair of residues. Next, these values were summed across all coevolving pairs yielding a coevolution strength for each pair of amino acids. To determine the extant to which these coevolution strengths diverged from random chance, the expected means and variances for the random variables representing random strengths were mathematically determined. A set of random strengths could be theoretically generated by randomly selecting residue pairs from the appropriate set (all, contacting, or distant) and repeating the calculation for determining the coevolution strengths but using the ZRes scores of the originally identified coevolving pairs as weights instead of the ZRes scores for the randomly selected sites. The final coevolution potential for each pair of amino acids was then calculated as the difference between their coevolution strength and the expected value of their random strength divided be the square root of the variance of their random strength.

### Annotation of Catalytic Sites

All representative structures utilized in the large-scale analysis were searched against the CSA database for catalytic residues [30]. Only those catalytic sites for which coevolution measures had been determined (i.e. those present in the Pfam alignment and containing <20% gaps) were analyzed. The CSA entries for all analyzed sites are provided in Table S3.

## Supporting Information

Figure S1 (0.11 MB PDF)

Figure S2 (0.14 MB PDF)

Figure S3 (0.23 MB PDF)

Figure S4 (0.17 MB PDF)

Figure S5 (0.11 MB PDF)

Figure S6 (0.12 MB PDF)

Table S1 (0.02 MB PDF)

Table S2 (0.04 MB PDF)

Table S3 (0.03 MB PDF)
