# Supplementary material for: Identification of Coevolving Residues and Coevolution Potentials Emphasizing Structure, Bond Formation and Catalytic Coordination in Protein Evolution
Source: PLoS One. 2009 Mar 10;4(3):e4762. doi: 10.1371/journal.pone.0004762 (PMC2651771; doi:10.1371/journal.pone.0004762)
Supplement: Figure S1 — (0.11 MB PDF) [file pone.0004762.s001.pdf]

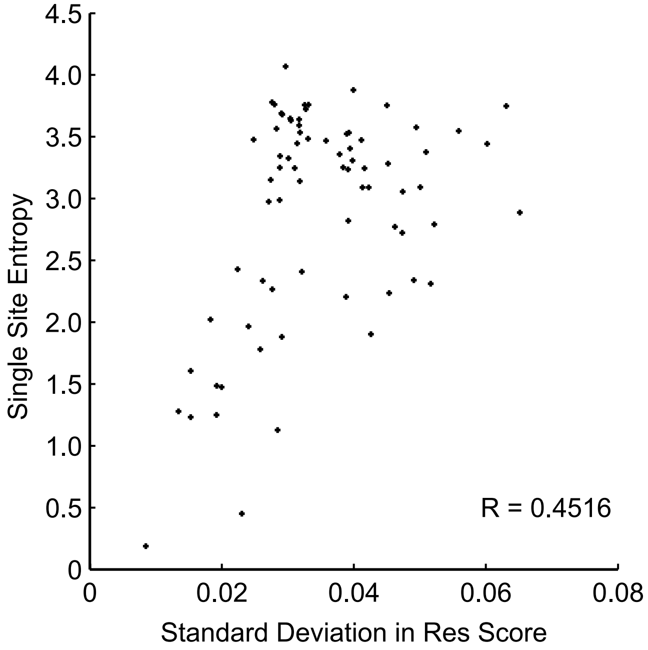

Figure S1. Within-site Res score variability correlates with entropy
The entropy of each site is plotted against the standard deviation of Res scores at that site. This suggests that sites with higher variation in amino acid composition are more likely to exhibit spuriously high Res scores when measured with multiple partner sites.
