# Supplementary material for: Identification of Coevolving Residues and Coevolution Potentials Emphasizing Structure, Bond Formation and Catalytic Coordination in Protein Evolution
Source: PLoS One. 2009 Mar 10;4(3):e4762. doi: 10.1371/journal.pone.0004762 (PMC2651771; doi:10.1371/journal.pone.0004762)
Supplement: Figure S2 — (0.14 MB PDF) [file pone.0004762.s002.pdf]

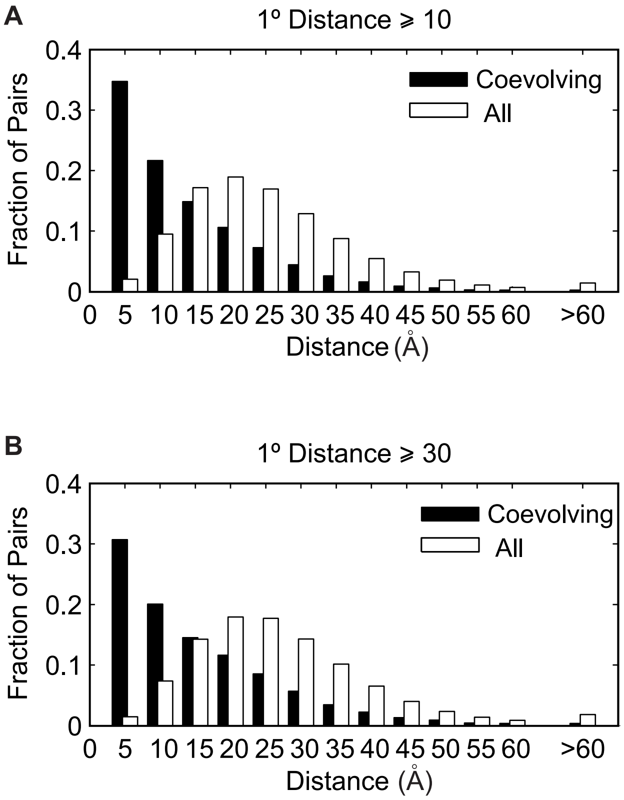

Figure S2. Distribution of distances between coevolving residues with minimal primary sequence separations

(A) The fraction of coevolving (black bars) or all (white bars) residue pairs with a primary distance of at least 10 amino acids that lie within the specified interval of physical distance from each other is depicted. (B) The fraction of coevolving (black bars) or all (white bars) residue pairs with a primary distance of at least 30 amino acids that lie within the specified interval of physical distance from each other is depicted.
