# Supplementary material for: Identification of Coevolving Residues and Coevolution Potentials Emphasizing Structure, Bond Formation and Catalytic Coordination in Protein Evolution
Source: PLoS One. 2009 Mar 10;4(3):e4762. doi: 10.1371/journal.pone.0004762 (PMC2651771; doi:10.1371/journal.pone.0004762)
Supplement: Figure S3 — (0.23 MB PDF) [file pone.0004762.s003.pdf]

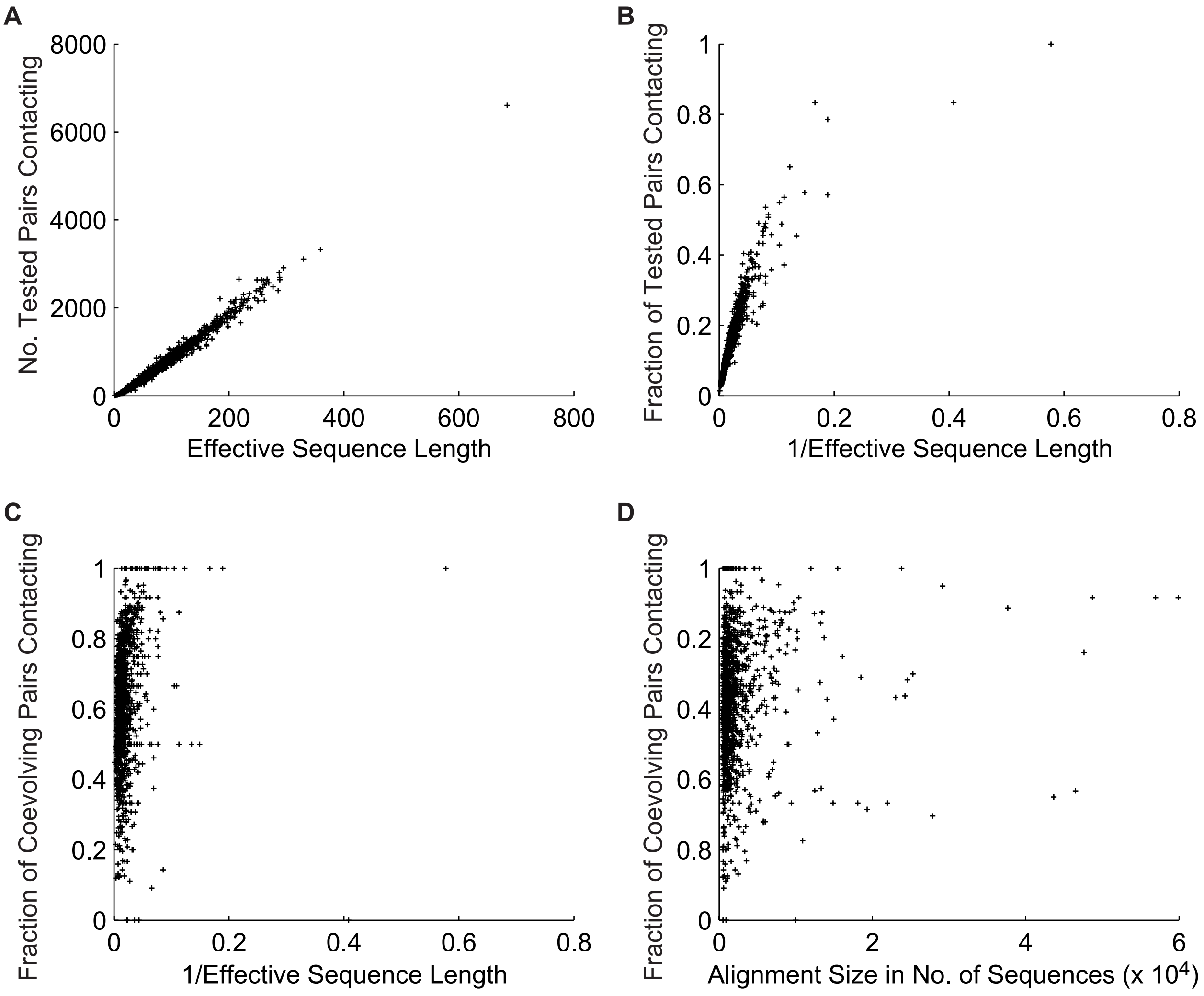


**Figure S3. Contact accuracy is weakly correlated with the reciprocal of protein length and alignment size.**

For each alignment for which a representative structure was available:
(A) The number of tested residue pairs that were contacting each other was plotted against the effective protein sequence length; (B) The fraction of the tested residue pairs that were contacting each other was plotted against the reciprocal of effective sequence length; (C) The fraction of residue pairs identified as coevolving that were contacting each other was plotted against the reciprocal of effective sequence length; (D) The fraction of residue pairs identified as coevolving that were contacting each other was plotted against the alignment size.
