# Supplementary material for: Identification of Coevolving Residues and Coevolution Potentials Emphasizing Structure, Bond Formation and Catalytic Coordination in Protein Evolution
Source: PLoS One. 2009 Mar 10;4(3):e4762. doi: 10.1371/journal.pone.0004762 (PMC2651771; doi:10.1371/journal.pone.0004762)
Supplement: Figure S4 — (0.17 MB PDF) [file pone.0004762.s004.pdf]

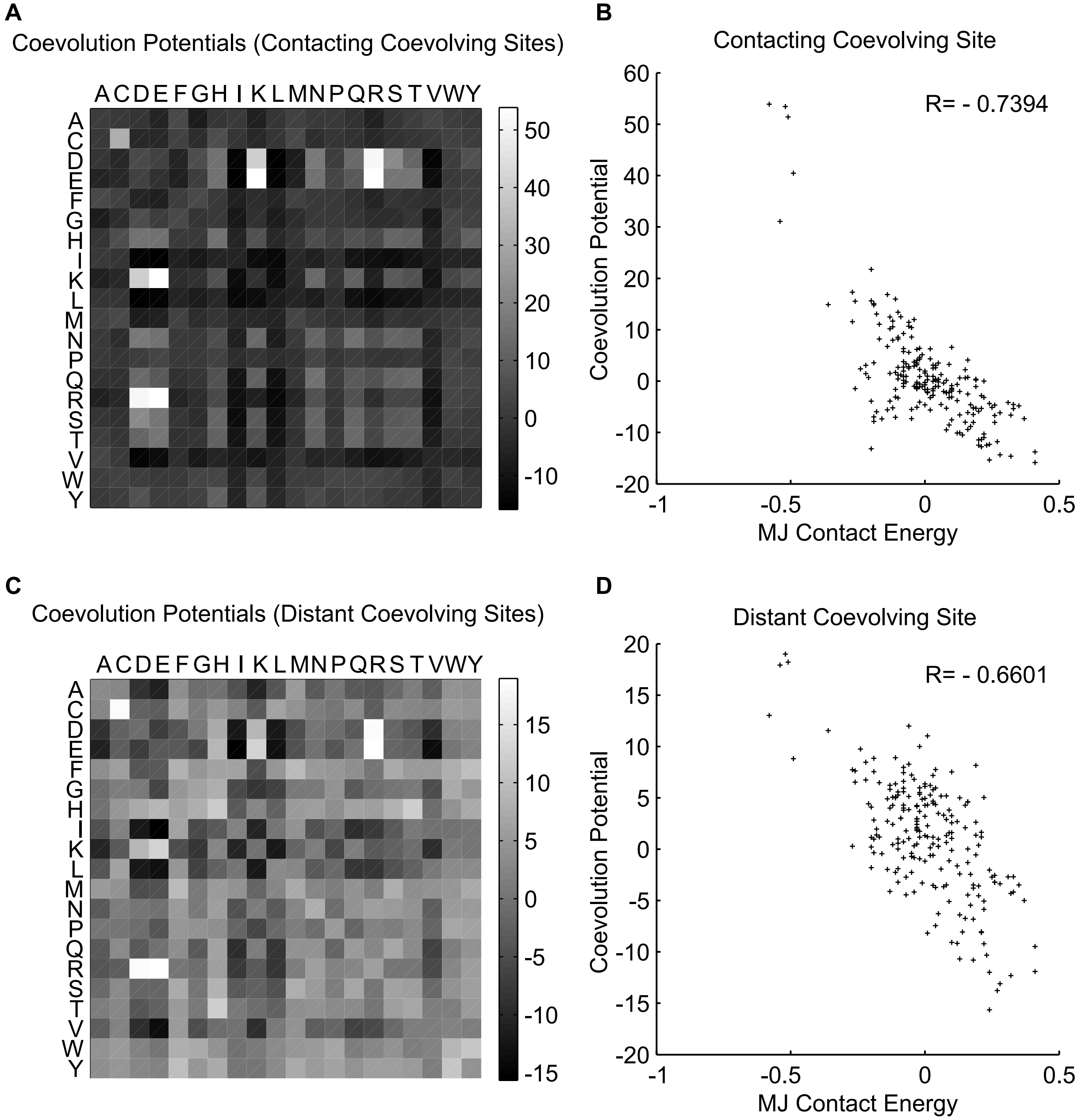

Figure S4. Amino acid coevolution potentials for contacting and distant residue pairs

(A) Coevolution potentials calculated only for residues pairs no further than 6 Å apart (intra-molecular distance). (B) Coevolution potentials amongst contacting residue pairs are correlated with the MJ contact energies. (C) Coevolution potentials calculated only for residues pairs that are at least 6 Å apart. (D) Coevolution potentials amongst distant residue pairs are still correlated with the MJ contact energies.
