# Supplementary material for: Identification of Coevolving Residues and Coevolution Potentials Emphasizing Structure, Bond Formation and Catalytic Coordination in Protein Evolution
Source: PLoS One. 2009 Mar 10;4(3):e4762. doi: 10.1371/journal.pone.0004762 (PMC2651771; doi:10.1371/journal.pone.0004762)
Supplement: Figure S5 — (0.11 MB PDF) [file pone.0004762.s005.pdf]

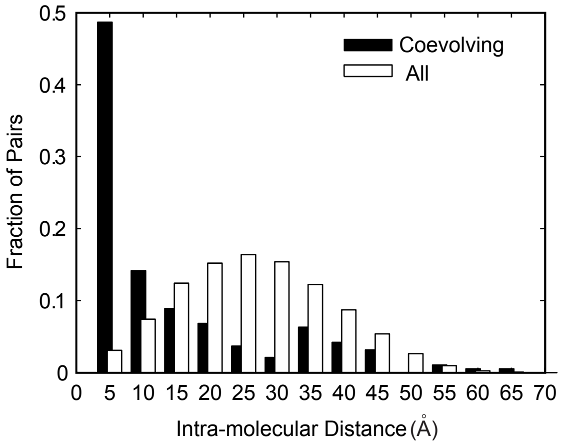

Figure S5. Distribution of intra-molecular distances between coevolving residues of chorismate synthase

The fraction of coevolving (black bars) or all (white bars) residue pairs that lie within the specified interval of physical distance from each other is depicted.
