# Supplementary material for: Identification of Coevolving Residues and Coevolution Potentials Emphasizing Structure, Bond Formation and Catalytic Coordination in Protein Evolution
Source: PLoS One. 2009 Mar 10;4(3):e4762. doi: 10.1371/journal.pone.0004762 (PMC2651771; doi:10.1371/journal.pone.0004762)
Supplement: Figure S6 — (0.12 MB PDF) [file pone.0004762.s006.pdf]

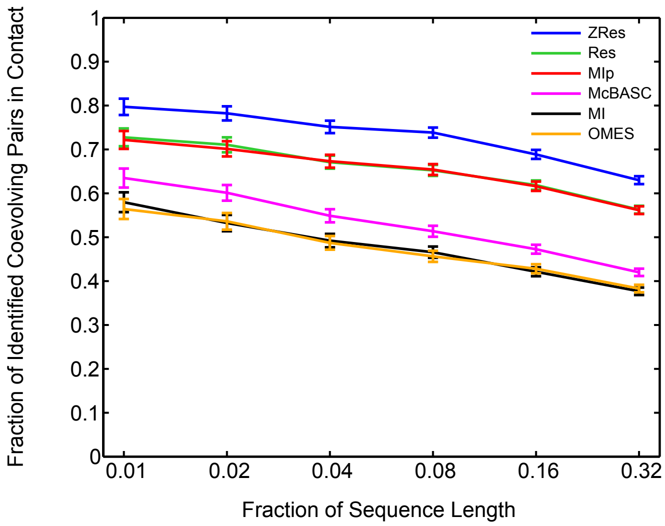

Figure S6. The same dataset used in Figure 9A was reanalyzed to normalize for variation in protein sequence length.

Again all residue pairs were ranked from highest to lowest ZRes score. For each protein, a number of top ranking residue pairs proportional to the length of the protein sequence (characterized as fractions of the protein sequence length) were considered. The fraction of these high-ranking residue pairs that lied within 6 Å of each other was then calculated. The average of this contact accuracy across all alignments was then plotted (blue). The process was repeated with the Res (green), OMES (brown), McBASC (magenta), MIp (red), and MI (black) measures.
