# Supplementary material for: Identification of Coevolving Residues and Coevolution Potentials Emphasizing Structure, Bond Formation and Catalytic Coordination in Protein Evolution
Source: PLoS One. 2009 Mar 10;4(3):e4762. doi: 10.1371/journal.pone.0004762 (PMC2651771; doi:10.1371/journal.pone.0004762)
Supplement: Table S1 — (0.02 MB PDF) [file pone.0004762.s007.pdf]

Table 1. Coevolution potentials between the 20 amino acids

All Coevolving Sites: Amino Acid Pair		Potential	Contacting Coevolving Sites: Amino Acid Pair		Potential	Distant Coevolving Sites: Amino Acid Pair		Potential
D	R	49.1968	E	K	53.9119	E	R	18.9854
C	C	46.1462	E	R	53.4425	D	R	18.2137
E	R	45.2460	D	R	51.4210	C	C	17.9298
E	K	44.2856	D	K	40.4732	E	K	13.0132
D	K	37.7880	C	C	31.0961	H	T	11.9935
H	H	33.1667	D	S	21.7523	H	H	11.5413
D	H	20.4930	D	N	17.2885	W	Y	11.0221
H	T	17.6948	E	S	16.8331	F	Y	9.9891
H	Y	17.4445	E	T	15.9794	F	M	9.7439
E	H	17.3740	N	Q	15.6016	W	W	8.9105
H	S	16.1707	D	H	15.5428	E	H	8.8520
V	V	14.2829	E	N	15.0940	D	K	8.8076
I	V	14.2073	H	H	14.8874	F	F	8.4565
T	T	12.8784	E	H	14.7974	H	S	8.2739
F	F	12.2937	D	Q	13.4029	M	S	8.1637
D	S	12.0181	K	N	13.0465	F	W	7.9653
D	N	11.9963	Q	T	12.5328	G	H	7.7594
H	N	11.2247	D	T	12.4891	N	N	7.7417
N	T	11.0682	Q	S	11.9890	D	H	7.6153
N	N	10.8739	N	S	11.6909	M	M	7.5499
N	Q	10.7698	N	N	11.5494	F	S	7.5298
A	V	10.6893	H	S	11.4270	M	Y	7.4575
F	Y	10.4630	K	Q	11.0681	H	Y	7.2114
C	H	10.2541	H	T	10.6838	G	W	7.1871
H	Q	9.7470	N	T	10.5469	Q	S	6.7900
W	W	9.7259	H	Y	10.4189	P	W	6.7368
N	S	9.6658	S	T	10.1887	T	T	6.6029
I	I	9.4088	T	T	9.2562	G	G	6.5297
Q	T	9.0025	K	S	8.5672	S	S	6.4664
S	T	8.9230	E	Q	8.5321	F	I	6.3643
S	S	8.3412	S	S	8.2059	F	H	6.3536
Q	S	8.1357	Q	Q	8.1865	P	Y	6.2307
Q	Q	7.7409	K	Y	7.9766	H	Q	6.2079
L	V	7.6471	Q	R	6.7386	C	L	6.1078
D	Y	7.5486	H	K	6.5793	C	F	5.9767
H	W	7.4132	K	T	6.3804	S	Y	5.9606
F	M	7.3229	N	R	6.3125	H	N	5.9286
F	W	7.2463	H	Q	6.2837	S	T	5.8209
M	M	7.1932	D	Y	6.1787	R	R	5.7738
E	N	6.9083	H	N	5.7483	N	R	5.5822
M	Y	6.8672	D	G	5.5752	R	Y	5.5766
D	T	6.8169	C	H	5.1441	H	R	5.5233
C	F	6.7458	H	R	4.6486	H	M	5.3513
M	V	6.7215	N	Y	4.3142	N	Y	5.2701
A	F	6.7171	A	F	4.2775	Q	Q	5.2622

A	A	6.6952	F	G	4.0836	T	Y	5.1821
N	Y	6.6266	C	M	4.0069	N	W	5.1659
K	N	6.5506	D	D	3.8009	C	W	5.1275
H	R	6.3679	R	S	3.7067	F	V	5.0943
A	M	6.2508	M	M	3.5681	N	S	5.0537
A	I	6.1056	R	W	3.5398	F	N	5.0357
Q	R	6.0562	K	W	3.5172	P	P	4.9960
W	Y	5.9252	A	V	3.3645	F	T	4.9929
R	Y	5.8784	Q	W	3.2690	A	M	4.9483
P	W	5.8755	R	T	3.1709	F	L	4.9030
P	Y	5.7523	G	W	3.0930	C	H	4.8572
F	V	5.7385	P	W	3.0590	G	Y	4.6665
K	Y	5.5717	E	P	3.0528	C	Y	4.6058
E	T	5.4346	N	P	2.9159	F	G	4.5963
D	Q	5.3880	Q	Y	2.8155	F	P	4.4893
K	Q	5.1300	H	W	2.7349	M	W	4.4170
Q	Y	4.9996	R	Y	2.6858	M	Q	4.3448
G	W	4.9899	D	E	2.6092	A	W	4.3035
C	V	4.9845	G	Y	2.5287	H	W	4.2890
C	W	4.8553	C	W	2.5178	Y	Y	4.2784
C	M	4.6747	F	M	2.3582	N	T	4.2629
G	Y	4.6648	C	F	2.2425	S	W	4.2614
H	M	4.6243	S	W	2.2171	M	V	4.2457
F	H	4.5303	A	M	1.9665	C	M	4.1523
I	M	4.2687	H	P	1.9556	N	Q	4.1044
G	H	4.2674	W	W	1.6774	G	P	4.0009
C	Y	4.2239	A	T	1.5575	I	M	3.9930
M	S	4.2205	P	Y	1.5033	Q	Y	3.8476
H	K	4.1740	F	F	1.4532	M	T	3.8197
M	T	4.1660	N	W	1.2513	H	P	3.8001
C	L	4.0306	P	Q	1.1752	A	F	3.6818
Q	W	4.0239	A	A	1.1731	A	Y	3.4924
H	P	3.9906	A	W	0.9409	F	Q	3.3103
L	L	3.9553	D	P	0.9237	C	Q	3.2547
Y	Y	3.9045	E	Y	0.9129	L	Y	3.2038
N	W	3.8191	F	Y	0.8921	A	A	3.0229
E	Q	3.7600	M	Y	0.8666	Q	T	3.0088
R	W	3.7158	C	Y	0.7151	L	W	2.9710
N	R	3.7115	M	W	0.6858	P	T	2.9471
T	Y	3.6599	E	G	0.6166	C	S	2.8994
F	G	3.6104	D	W	0.5969	L	L	2.8266
F	L	3.3760	G	N	0.4374	Q	R	2.7218
A	W	3.2583	G	H	0.3993	R	W	2.7144
A	C	3.1696	T	W	0.3911	C	T	2.6741
M	W	2.8700	A	P	0.3459	M	N	2.6307
C	I	2.7730	E	W	0.3105	H	K	2.5742
E	S	2.6892	A	C	0.2808	L	M	2.4193
S	Y	2.6511	M	N	0.2067	D	Y	2.4189
A	L	2.6021	G	P	0.1620	A	C	2.2692

A	Y	2.5596	S	Y	0.1492	C	I	2.1343
K	T	2.3384	T	Y	0.1365	D	W	2.0892
S	W	2.2492	F	N	0.0106	P	R	1.9650
T	V	2.1888	F	P	-0.0288	I	I	1.9525
F	N	2.1590	A	I	-0.1501	C	V	1.8121
G	G	2.0624	A	Y	-0.1578	E	Y	1.7812
F	P	2.0617	C	V	-0.2281	V	W	1.7492
T	W	1.6863	Y	Y	-0.2490	M	R	1.6410
F	I	1.5346	F	H	-0.2624	T	V	1.6020
V	Y	1.5330	A	N	-0.3815	Q	W	1.5860
M	N	1.4977	F	W	-0.4484	K	N	1.3825
A	T	1.4484	P	T	-0.5646	T	W	1.3501
D	G	1.3389	P	S	-0.5835	A	T	1.3260
M	Q	1.2897	C	T	-0.6407	V	Y	1.3173
K	S	1.1993	A	S	-0.7411	M	P	1.2156
R	S	1.0174	A	H	-0.8919	C	N	1.1966
D	W	1.0034	A	L	-1.0022	K	Q	1.1940
K	W	0.9949	K	P	-1.0244	C	G	1.1753
L	Y	0.9112	M	Q	-1.0258	P	S	1.1697
I	L	0.7500	G	T	-1.0390	V	V	1.1490
F	S	0.4812	G	Q	-1.0673	G	M	1.0511
L	M	0.4439	M	S	-1.0853	D	G	1.0408
P	P	0.4004	H	M	-1.1086	G	N	0.9874
R	R	0.3661	M	V	-1.2243	K	W	0.9740
F	T	-0.0313	A	D	-1.3647	L	V	0.9677
E	Y	-0.1091	P	R	-1.4247	G	T	0.7163
N	P	-0.1131	G	G	-1.4598	I	T	0.6506
F	Q	-0.2795	C	P	-1.4870	G	S	0.5660
C	T	-0.4166	C	I	-1.4988	C	P	0.5101
C	Q	-0.5385	P	P	-1.6103	R	T	0.4990
R	T	-0.5625	W	Y	-1.6143	K	Y	0.4327
D	D	-0.6434	F	S	-1.6550	F	R	0.3804
I	T	-0.6806	C	L	-1.6617	R	S	0.3196
A	H	-0.7578	E	E	-1.8778	D	N	0.2960
C	G	-1.0737	C	Q	-1.9564	E	W	0.2618
I	Y	-1.1034	F	Q	-2.0282	A	P	0.2617
G	P	-1.1405	F	T	-2.0395	I	L	0.2196
E	W	-1.1546	G	K	-2.0984	I	Y	0.1285
L	W	-1.1757	C	S	-2.2197	E	Q	0.0138
V	W	-1.2772	M	T	-2.2258	K	K	0.0113
P	T	-1.3144	M	P	-2.2705	A	H	-0.1061
D	P	-1.3417	A	Q	-2.3018	E	N	-0.3512
G	N	-1.7356	K	K	-2.5198	I	W	-0.4001
C	S	-2.1627	C	G	-2.7751	I	V	-0.4466
C	P	-2.3834	R	R	-2.8034	H	V	-0.5508
P	Q	-2.4516	C	N	-2.8271	D	P	-0.5867
K	K	-2.4619	G	M	-3.0941	G	R	-0.6193
C	N	-2.5744	F	V	-3.1064	A	G	-0.7006
M	P	-2.6183	F	K	-3.1939	N	P	-0.8258

H	V	-2.7153	G	R	-3.2021	D	D	-0.8501
G	M	-3.2387	L	P	-3.2166	K	T	-0.9478
G	T	-3.2682	G	S	-3.2840	L	P	-1.1306
A	Q	-3.3428	I	P	-3.7866	A	S	-1.1709
A	S	-3.4066	V	V	-3.8907	S	V	-1.3073
P	S	-3.5302	I	M	-3.9020	I	P	-1.6247
A	N	-3.6461	V	W	-4.0979	D	S	-1.8001
M	R	-3.7407	C	E	-4.1908	P	Q	-1.8866
K	M	-3.7891	K	M	-4.3667	P	V	-1.9268
I	W	-3.8198	P	V	-4.5496	E	S	-1.9704
H	L	-3.9419	C	R	-4.5712	K	M	-2.0277
H	I	-4.1357	H	L	-4.6096	E	T	-2.2536
S	V	-4.6430	L	Y	-4.6152	K	S	-2.2609
L	P	-5.0441	C	D	-4.6849	L	T	-2.4192
P	R	-5.0781	C	K	-4.8078	H	L	-2.4502
E	P	-5.1370	L	W	-5.0769	C	D	-2.5509
N	V	-5.3402	N	V	-5.1160	C	R	-2.6771
I	N	-5.4984	L	M	-5.2122	C	E	-2.6984
G	S	-5.6653	A	E	-5.2209	A	V	-2.7205
A	P	-5.6712	H	I	-5.2512	N	V	-2.7220
F	R	-5.6917	V	Y	-5.2701	E	F	-3.2153
E	E	-5.7902	H	V	-5.2795	D	Q	-3.2234
C	R	-5.8784	M	R	-5.3386	L	N	-3.2255
C	D	-6.2569	D	F	-5.3638	G	Q	-3.2951
I	S	-6.4008	I	W	-5.4942	K	R	-3.3789
I	P	-6.5084	F	L	-5.5754	A	Q	-3.4760
E	M	-6.8368	I	Y	-5.6121	H	I	-3.4819
D	F	-6.8922	F	R	-5.9117	C	K	-3.4880
G	Q	-6.9845	I	I	-5.9668	E	E	-3.5180
C	E	-7.1196	E	M	-6.1059	I	S	-3.5712
P	V	-7.2956	K	R	-6.1543	A	N	-3.6655
D	M	-7.2980	A	R	-6.5078	E	P	-3.7401
K	R	-7.3517	A	K	-6.5763	A	R	-3.7904
Q	V	-7.4373	I	N	-6.6382	D	T	-4.1121
G	R	-7.7431	E	F	-6.6796	A	L	-4.1723
D	E	-8.0676	L	V	-6.9747	D	F	-4.1724
F	K	-8.0993	F	I	-7.0293	I	N	-4.3041
L	T	-8.1147	A	G	-7.2962	A	I	-4.4400
C	K	-8.4225	D	M	-7.3260	E	M	-4.4696
K	P	-8.9758	I	V	-7.3495	L	S	-4.5393
A	R	-9.6193	T	V	-7.5417	D	M	-5.0036
E	F	-9.7233	L	L	-7.8385	F	K	-5.0504
I	Q	-9.8229	Q	V	-8.0055	G	V	-5.0913
L	N	-9.8586	G	L	-8.0456	G	I	-5.4516
A	D	-10.0463	L	N	-8.0464	R	V	-5.8639
I	R	-10.7521	G	V	-8.5373	K	P	-6.2789
E	G	-11.3221	G	I	-8.8373	E	G	-6.3896
K	V	-11.4676	S	V	-9.4255	Q	V	-6.7407
R	V	-11.4993	I	T	-9.5049	G	L	-6.8245

L	Q	-11.6831	L	T	-10.0827	D	E	-7.4417
A	G	-12.1519	I	Q	-10.1114	I	R	-8.0456
G	K	-12.1685	K	V	-10.2953	L	Q	-8.0644
A	K	-12.1724	L	Q	-10.4772	L	R	-8.1170
L	S	-12.8170	R	V	-10.9947	G	K	-8.1811
G	I	-13.0582	I	R	-11.3638	A	D	-9.0713
G	V	-13.2751	L	S	-11.4346	I	Q	-9.1635
I	K	-14.2028	E	V	-11.7883	K	V	-9.2044
A	E	-14.3971	K	L	-12.3117	D	V	-9.4837
L	R	-15.2018	I	S	-12.4610	I	K	-10.3179
G	L	-16.0751	I	K	-12.4771	A	K	-10.6924
K	L	-16.5458	L	R	-12.7793	A	E	-10.7913
D	V	-16.5582	I	L	-13.1824	D	L	-11.9101
D	I	-17.3772	D	V	-13.7751	K	L	-11.9795
E	V	-18.9170	E	L	-14.3680	D	I	-12.3050
E	I	-20.0630	D	I	-14.6211	E	L	-13.0887
E	L	-20.5412	E	I	-15.3509	E	V	-13.7622
D	L	-21.8957	D	L	-15.8368	E	I	-15.6352
