# Supplementary material for: Identification of Coevolving Residues and Coevolution Potentials Emphasizing Structure, Bond Formation and Catalytic Coordination in Protein Evolution
Source: PLoS One. 2009 Mar 10;4(3):e4762. doi: 10.1371/journal.pone.0004762 (PMC2651771; doi:10.1371/journal.pone.0004762)
Supplement: Table S2 — (0.04 MB PDF) [file pone.0004762.s008.pdf]

Table 2. List of analyzed Pfam alignments, representative crystal structures, and the sequence numbers for these structures in the alignments

Pfam ID	PDB ID	Seq. #
PF00385	2b2t	87
PF02461	1yew	2485
PF00306	1bmf	4453
PF02790	1m56	9906
PF01510	1aro	776
PF00166	1aon	143
PF06144	1jqj	357
PF00510	1fft	3737
PF01638	1yyv	939
PF00889	1aip	263
PF00036	1a03	4269
PF01012	1efp	130
PF00941	1ffu	59
PF07554	1xvh	474
PF01346	1fd9	127
PF01468	1prb	623
PF01162	1zq1	581
PF02934	1zq1	598
PF02347	1wyt	554
PF00517	1aik	5165
PF01966	2paq	4493
PF00529	1t5e	157
PF02022	1e0e	1972
PF03641	1t35	62
PF02249	1e6v	1067
PF00206	1aos	1361
PF01795	1m6y	321
PF00244	1a37	13
PF00389	1dld	2096
PF02826	1dld	1906
PF00198	1b5s	895
PF02834	1vdx	261
PF03171	1bk0	1912
PF01612	1d8y	138
PF00725	1f0y	790
PF02737	1f0y	245
PF03061	1bvq	3084
PF01812	1ydm	63
PF01367	1bgx	278
PF02739	1bgx	527
PF02872	1ho5	582
PF02096	----	----
PF00393	1pgj	982
PF00001	1l4t	2083
PF00002	1bl1	163
PF01461	----	----
PF00962	1a4l	214
PF00696	1b7b	1492

PF00324	----	----
PF01490	----	----
PF00004	1d2n	7242
PF07724	1do0	3059
PF07726	----	----
PF07728	1hn5	1835
PF00950	----	----
PF03109	----	----
PF01061	----	----
PF00664	----	----
PF00561	1a7u	3628
PF02230	1auo	80
PF07859	1evq	306
PF02517	----	----
PF03992	1sqe	282
PF00871	1g99	134
PF00583	1b6b	8154
PF00328	1cvi	95
PF00330	1aco	1493
PF00694	1aco	710
PF08545	1ebl	24
PF08541	1ebl	153
PF01648	1f7l	242
PF00873	1iwg	1191
PF01842	1in9	5762
PF00441	1buc	339
PF08028	----	----
PF02770	1buc	1189
PF02771	1buc	913
PF02551	1tbu	322
PF00698	1mla	147
PF01757	----	----
PF00708	1gxt	501
PF01553	1iuq	2561
PF00608	1qiu	158
PF00709	1ade	319
PF08240	1a71	2035
PF00106	1a27	9687
PF00107	1a71	5869
PF00406	1ak2	93
PF05191	1ak2	385
PF05221	1a7a	122
PF00670	1a7a	204
PF02420	1ezg	7
PF00578	1e2y	1605
PF08327	1z94	388
PF01808	1g8m	608
PF00731	1xmp	221
PF00586	1t3t	2128
PF02769	1cli	2397
PF00842	1bd0	22
PF01168	1b54	961
PF00490	1aw5	615
PF01262	1pjb	601

PF05222	1pjb	150
PF01315	1dgj	853
PF00171	1a4s	955
PF00248	1a80	2891
PF00596	1dzu	593
PF01263	1jov	690
PF00245	1aja	59
PF00128	1a47	3228
PF02806	1a47	539
PF00943	----	----
PF01425	1m21	1348
PF01520	1jwq	126
PF04909	2ffi	131
PF07969	1k6w	874
PF01593	1b37	1718
PF00155	1aam	123
PF00202	1d7r	2536
PF01063	1a0g	907
PF00266	1bjn	3077
PF00909	1xqe	547
PF00501	1amu	5038
PF01094	1ewk	68
PF00023	1a5e	47065
PF00191	1avc	1626
PF04715	1i1q	824
PF08264	1ffy	1842
PF00847	1gcc	43
PF01261	1a0c	1944
PF02558	2ew2	73
PF08546	2ew2	360
PF01636	1j7i	856
PF01583	1d6j	264
PF02311	1xja	616
PF06445	1d5y	585
PF00025	1e0s	317
PF01412	1dcq	278
PF03485	1bs2	673
PF00491	1cev	506
PF00764	1j1z	390
PF00514	1bk5	387
PF03960	1i9d	109
PF00951	----	----
PF00733	1ct9	249
PF01037	1i1g	2145
PF01177	1b73	181
PF00710	1agx	83
PF02178	2ezd	1425
PF03477	1r1r	155
PF02222	1b6r	864
PF08442	1euc	50
PF00217	1bg0	672
PF02807	1bg0	642
PF00231	1bmf	660
PF00895	----	----

PF00006	1bmf	6185
PF02874	1bmf	4473
PF00430	1b9u	138
PF00137	1a91	1383
PF00401	1aqt	228
PF02823	1aqt	364
PF01171	1ni5	385
PF03797	1uyn	31
PF02310	1b1a	812
PF02607	1bmt	42
PF02362	1wid	1
PF03483	1b70	230
PF03484	1b70	82
PF01453	1b2p	128
PF00216	1ihf	877
PF00308	1l8q	623
PF08299	1j1v	43
PF01312	----	----
PF00296	1brl	1883
PF01103	----	----
PF02397	----	----
PF01654	----	----
PF02673	----	----
PF07707	----	----
PF01145	1lu7	1536
PF06968	1r30	61
PF02028	----	----
PF01869	1zc6	505
PF00407	1b6f	100
PF01212	1ax4	45
PF02369	1cwv	540
PF02368	1e5u	900
PF02785	1bnc	235
PF00364	1a6x	1317
PF00653	1c9q	159
PF01766	1wcd	1056
PF00936	2a10	246
PF02012	1dil	1305
PF01722	1v60	11
PF04972	----	----
PF00528	----	----
PF02653	----	----
PF03099	1bia	192
PF00533	1jnx	1882
PF00439	1jsp	814
PF00651	1r28	326
PF03401	2f5x	1282
PF00170	1dh3	343
PF07716	1gtw	360
PF00130	1xa6	240
PF07649	----	----
PF00168	1a25	357
PF08205	1pwi	118
PF02363	----	----

PF02743	----	----
PF00028	1ncj	3055
PF08266	1wyj	102
PF03507	----	----
PF07337	----	----
PF00915	1ihm	1127
PF03160	----	----
PF00194	12ca	356
PF01256	1kyh	356
PF01039	1od2	1724
PF02559	----	----
PF00199	1a4e	827
PF00689	1fqu	310
PF00690	1fqu	325
PF01545	----	----
PF00808	1n1j	299
PF01656	1a82	2728
PF02361	----	----
PF01607	1dqc	563
PF02839	1aiw	368
PF00571	1b3o	1702
PF02754	----	----
PF01066	----	----
PF00150	1a3h	611
PF00307	1rt8	601
PF02797	1bi5	655
PF00195	1bi5	340
PF05257	----	----
PF01339	1a2o	52
PF04509	1squ	401
PF01739	1af7	29
PF03705	1af7	86
PF01584	1k0s	860
PF00379	----	----
PF01308	----	----
PF02415	----	----
PF00504	1rwt	100
PF00425	1i1q	1053
PF01264	1q1l	581
PF02417	----	----
PF02464	----	----
PF06022	----	----
PF03600	----	----
PF00285	1a59	1397
PF01217	2vgl	44
PF00637	1b89	313
PF01785	----	----
PF02861	1k6k	1847
PF00574	1r8z	962
PF01817	1csm	737
PF02353	1kp9	489
PF02627	2af7	274
PF00795	1ems	1036
PF05738	1d2o	1863

PF00027	1apk	1216
PF02629	1euc	158
PF02515	1p5h	1601
PF01121	1jjv	278
PF02492	1nij	772
PF07683	1nij	151
PF03772	----	----
PF01257	----	----
PF00329	----	----
PF00346	----	----
PF01512	----	----
PF00668	1q9j	3587
PF03602	1wxw	36
PF01544	2bbh	264
PF03471	----	----
PF01600	----	----
PF01601	1wnc	245
PF00116	1cyw	11462
PF00118	1a6d	5034
PF00289	1a9x	254
PF02786	1a9x	892
PF02787	1a9x	22
PF00988	1a9x	474
PF00650	1aua	220
PF02537	----	----
PF01321	1chm	348
PF00325	1cgp	180
PF00030	1a45	194
PF04969	1x5m	162
PF00313	1c9o	467
PF06418	1s1m	460
PF01148	----	----
PF01467	1b6t	774
PF02348	1eyr	534
PF00394	1a65	2233
PF07731	1a65	256
PF07732	1a65	681
PF00431	1nt0	1281
PF00190	1cau	290
PF07883	1o4t	1092
PF07012	----	----
PF01473	1gvm	1795
PF02984	1e9h	179
PF00134	1bu2	705
PF00007	1hcn	275
PF01053	1cl1	106
PF00031	1a67	64
PF00173	1aw3	53
PF02224	1q3t	255
PF02322	----	----
PF00283	1s5l	23
PF00032	1bcc	12258
PF00033	1bcc	17779
PF01578	----	----

PF02239	1aof	1637
PF00781	----	----
PF00793	1d9e	227
PF07478	1e4e	538
PF01820	1e4e	588
PF05746	1bs2	656
PF09190	1li5	97
PF01266	1an9	6277
PF01678	1bwz	624
PF05173	1arz	236
PF01113	1arz	43
PF00383	1af2	354
PF06808	----	----
PF04290	----	----
PF00270	1fuu	3308
PF01041	1b9h	449
PF02645	1pzx	76
PF01791	1j2w	497
PF00455	1lk5	696
PF04127	1p9o	562
PF00926	1g57	473
PF00701	1dhp	295
PF00186	1ai9	680
PF01368	1ir6	467
PF02272	1ir6	1014
PF01180	1d3g	198
PF01761	1dqs	399
PF00775	1dlm	237
PF00200	1l3x	126
PF04977	----	----
PF01965	1g2i	662
PF01738	1ggv	332
PF00885	1c2y	292
PF01035	1eh6	630
PF03989	1suu	4928
PF00204	1kij	3235
PF00986	----	----
PF04679	1x9n	91
PF01068	1a0i	799
PF01653	1b04	243
PF03120	1dgs	167
PF03119	1dgs	6
PF01119	1ea6	608
PF00875	1dnp	397
PF07733	----	----
PF00712	2avt	551
PF02767	2avt	481
PF02768	2avt	194
PF00476	1bgx	289
PF03104	1clq	571
PF00336	----	----
PF00242	----	----
PF00772	1b79	274
PF03796	----	----

PF00226	1fpo	5068
PF01556	1nlt	141
PF00684	1nlt	122
PF00404	1daq	40
PF07681	----	----
PF03330	1n10	359
PF01323	1bed	203
PF02683	----	----
PF00782	1d5r	867
PF00035	1uil	1538
PF00908	1dzr	71
PF06429	----	----
PF01345	----	----
PF02405	----	----
PF02410	----	----
PF02575	1j8b	113
PF07564	----	----
PF07717	----	----
PF02588	----	----
PF08330	----	----
PF01595	----	----
PF02698	----	----
PF03193	1t9h	61
PF01657	----	----
PF01709	1mhf	624
PF03703	----	----
PF03781	1y1e	301
PF04143	----	----
PF01882	----	----
PF04524	----	----
PF01883	1uwd	333
PF00892	----	----
PF01925	----	----
PF05685	----	----
PF06071	1jal	230
PF06114	----	----
PF05424	2c6j	2805
PF01207	1vhn	740
PF00692	1duc	762
PF00350	1jwy	483
PF00122	1fqu	2786
PF00676	1dtw	1339
PF02817	1ebd	945
PF00563	2bas	4044
PF00378	1dci	3495
PF00679	1dar	388
PF03764	1dar	88
PF01132	1ueb	333
PF08207	1ueb	484
PF00008	1a3p	6050
PF07974	1lh9	1905
PF07645	1adx	2359
PF00971	----	----
PF01176	1ah9	285

PF03610	1pdo	89
PF00208	1aup	286
PF02812	1aup	814
PF03789	----	----
PF09285	1ueb	103
PF00113	1e9i	219
PF03952	1e9i	880
PF01370	1a9y	2333
PF02350	1f6d	378
PF00275	1a2n	1146
PF03734	1y7m	783
PF00756	1pv1	238
PF00766	1efp	280
PF00178	1pue	506
PF02472	----	----
PF03372	1ako	1862
PF02601	----	----
PF00929	1fxx	1468
PF06508	----	----
PF02095	----	----
PF00646	1nex	2657
PF00469	1qa4	20
PF03807	1i36	1160
PF01116	1b57	704
PF00487	----	----
PF04116	----	----
PF01557	1gtt	1054
PF07977	1mka	671
PF02913	1ahu	832
PF00667	1amo	349
PF00890	1chu	193
PF01494	1bf3	750
PF01565	1ahu	1788
PF00970	1a8p	1848
PF03441	1dnp	398
PF06574	1mrz	549
PF04385	----	----
PF01149	1ee8	230
PF01581	----	----
PF02469	1o70	877
PF07729	----	----
PF03147	1b70	207
PF00465	1jpu	1416
PF01521	1nwb	479
PF01032	1l7v	624
PF04023	1fx7	8
PF00111	1a70	138
PF01799	1dgj	334
PF04324	1y56	928
PF00037	1a6l	1549
PF00142	1de0	2996
PF00373	1e5w	477
PF09379	1e5w	276
PF01794	----	----

PF00210	1z6o	584
PF00762	1ak1	15
PF01839	1jv2	109
PF02782	1bo5	841
PF00370	1bo5	287
PF00498	1dmz	1789
PF00771	----	----
PF00147	1fza	463
PF02661	----	----
PF05594	1rwr	2106
PF00630	1qfh	957
PF00419	1o9v	21
PF00254	1a7x	644
PF00700	1io1	239
PF07196	----	----
PF00669	1ucu	1001
PF07652	2bhr	352
PF02832	1pjw	214
PF01004	----	----
PF00948	----	----
PF01005	----	----
PF01002	----	----
PF01349	----	----
PF00972	1l9k	206
PF01570	----	----
PF01613	1i0r	685
PF00258	1ag9	128
PF02525	1d4a	239
PF01687	1q9s	556
PF02441	1E+20	558
PF00286	----	----
PF00460	----	----
PF09479	----	----
PF00813	----	----
PF01698	----	----
PF00598	1aa7	3572
PF08289	----	----
PF00506	1hoc	2672
PF00600	1lcq	6606
PF00601	1lcq	3384
PF00602	1wbz	3410
PF00604	----	----
PF04205	----	----
PF01070	1al8	278
PF03358	1rtt	271
PF00039	1fbr	52
PF00041	1uem	4397
PF05725	----	----
PF02152	1b9l	491
PF00250	1d5v	375
PF02911	1fmt	119
PF00551	1c2t	966
PF01268	1eg7	472
PF02491	1e4f	982

PF01728	1l9k	978
PF09397	----	----
PF01580	----	----
PF01098	----	----
PF02687	----	----
PF04082	----	----
PF01475	1mzb	210
PF01363	1hyi	107
PF01585	----	----
PF02781	1dpg	916
PF00479	1dpg	317
PF01019	----	----
PF02938	1c0a	64
PF01590	1vhm	1853
PF08705	1m4p	1256
PF00337	1c1f	404
PF01071	1gso	153
PF02843	1gso	95
PF02844	1gso	178
PF00320	1gat	494
PF00117	1a9x	2463
PF00310	1ao0	1032
PF07685	----	----
PF02637	1zq1	538
PF07670	----	----
PF01597	1dxm	118
PF01571	1pj5	483
PF08669	1pj5	783
PF03009	1o1z	266
PF00626	1c0f	388
PF00799	1l2m	679
PF08283	----	----
PF01440	----	----
PF01407	----	----
PF01492	----	----
PF00844	----	----
PF01524	----	----
PF00196	1fse	3350
PF04828	1x6m	462
PF01408	1evj	1607
PF02894	1evj	774
PF00990	1w25	2938
PF08544	1fi4	1692
PF00288	1fi4	610
PF01134	----	----
PF02527	1xdz	127
PF08388	----	----
PF01541	1ln0	771
PF00120	1f1h	316
PF03951	1f1h	584
PF03710	1v4a	86
PF00042	101m	1279
PF01645	1ea0	251
PF01182	1cd5	241

PF01832	----	----
PF07581	----	----
PF00462	1a8l	1279
PF01228	1cm5	197
PF00232	1bga	958
PF00331	1b30	13
PF00332	1aq0	541
PF00704	1c3f	1825
PF00182	1cns	91
PF00703	1bgl	592
PF00295	1bhe	850
PF02836	1bgl	105
PF02837	1bgl	188
PF00933	1ex1	751
PF01055	1xsi	468
PF08244	1st8	106
PF00251	1st8	121
PF01915	1ex1	610
PF04101	1f0k	453
PF03033	1f0k	377
PF00777	----	----
PF08323	1rzu	1085
PF01501	1g9r	268
PF01075	1psw	614
PF00274	1a5c	129
PF00802	1brv	1735
PF02885	1azy	828
PF00534	2bfw	7386
PF00535	1xhb	5960
PF00591	1azy	741
PF00953	----	----
PF00903	1bh5	5
PF05199	1cf3	951
PF00732	1cf3	865
PF00958	1gpm	151
PF00392	1e2x	5954
PF00516	1niz	26800
PF02800	1a7k	121
PF00044	1a7k	1764
PF00746	----	----
PF01272	2eul	698
PF03449	1grj	84
PF00255	1gp1	725
PF00437	1g6o	1573
PF05157	2d27	328
PF00482	----	----
PF00043	11gs	3394
PF02798	11gs	3885
PF01018	1lnz	350
PF00925	2bz0	271
PF01227	1a8r	845
PF00009	1aip	9825
PF03144	1aip	10586
PF03143	1aip	258

PF00211	1ab8	1239
PF00625	1s4q	495
PF01493	1ea0	435
PF02895	1b3q	38
PF06831	1ee8	38
PF04408	----	----
PF01725	1k7k	275
PF00672	1h2s	4312
PF02184	----	----
PF02518	1a4h	20413
PF01543	1cwx	587
PF01542	----	----
PF01560	----	----
PF01538	----	----
PF01001	----	----
PF01506	1r7c	1585
PF08300	1zh1	312
PF08301	1zh1	13
PF08668	----	----
PF01517	1a92	378
PF05345	----	----
PF02985	1b3u	2666
PF03130	1te4	460
PF00632	1c4z	14
PF00271	1a1v	7964
PF02602	1wcw	558
PF01814	1a7d	1302
PF06969	1olt	20
PF00353	1af0	1283
PF00045	1ck7	405
PF08290	----	----
PF05658	1p9h	198
PF06985	----	----
PF00132	1fwy	3730
PF03129	1adj	2222
PF03578	----	----
PF00633	1ebm	862
PF00730	1diz	2114
PF03486	----	----
PF05662	1p9h	720
PF00977	1gpw	440
PF06580	----	----
PF01634	1h3d	12
PF00512	2c2a	6172
PF07730	----	----
PF00850	1zz0	454
PF00815	1k75	421
PF00125	1aoi	308
PF01230	1av5	188
PF00010	1a0a	1246
PF03006	----	----
PF00403	1afi	1183
PF00505	1aab	2057
PF00682	1nvm	1209

PF01844	1bxi	1292
PF00046	1ahd	1915
PF00742	1ebf	483
PF00104	1rdt	552
PF03328	1dxe	636
PF01288	1cbk	51
PF01627	1i5n	1165
PF00570	1wud	203
PF04313	----	----
PF00011	1gme	1184
PF00126	1b9m	13511
PF08279	1i1g	1186
PF01381	1b0n	1953
PF01022	1ri7	2748
PF01418	----	----
PF02796	1hcr	398
PF02954	1etk	1628
PF00165	1adn	7141
PF08220	----	----
PF09339	1mkm	1742
PF01968	----	----
PF00702	1aq6	9690
PF08282	1l6r	1217
PF07679	1fhg	3767
PF03810	1f59	253
PF01485	1wim	428
PF00818	1ina	511
PF00463	1dqu	253
PF01614	1mkm	280
PF01008	1t5o	205
PF04760	1nd9	9
PF00707	2crq	593
PF05198	1tif	28
PF00047	1als	5661
PF00475	1rhy	498
PF00218	1a53	1203
PF00048	1f2l	255
PF01450	1np3	16
PF00920	----	----
PF07991	1np3	159
PF00478	1ak5	937
PF00817	1im4	827
PF08246	1by8	508
PF00459	1awb	518
PF00552	1c0m	2140
PF01348	----	----
PF00904	1eu0	235
PF07885	1bl8	650
PF01715	----	----
PF00612	1aji	994
PF00180	1a05	624
PF02922	1bf2	321
PF00857	1ilw	79
PF01128	1fwy	445

PF01695	----	----
PF01156	1ezr	251
PF02373	----	----
PF01486	----	----
PF02214	1a68	594
PF00050	1bmo	15
PF07648	1an1	216
PF01344	1gof	3420
PF07646	----	----
PF00109	1b3n	3563
PF02801	1b3n	3640
PF00013	1hh2	3341
PF07650	1ega	1199
PF02524	----	----
PF00225	1bg2	567
PF03790	----	----
PF03791	----	----
PF00467	1m1g	954
PF08659	----	----
PF01352	1v65	1096
PF00051	1a0h	636
PF00014	1aal	415
PF00256	1gs2	193
PF02502	1nn4	108
PF00356	1bdh	1351
PF00753	1a7t	3640
PF00961	1m5x	350
PF00053	1klo	1875
PF02210	1c4r	1025
PF00057	1jrf	1765
PF00058	1ijq	44
PF00059	1g1s	1336
PF06421	----	----
PF08502	1sr9	607
PF01790	----	----
PF00549	1euc	799
PF00412	1g47	2783
PF00538	1uss	178
PF03279	----	----
PF01764	1usw	133
PF00657	1bwp	956
PF00061	1bm5	421
PF03180	1p99	410
PF01451	1bvh	666
PF02190	1zbo	718
PF05362	1qzm	126
PF00560	1a4y	32590
PF07723	1fqv	479
PF01462	1m0z	1548
PF08263	1ogq	111
PF01423	1b34	1087
PF00677	1kzl	1024
PF00894	----	----
PF01810	----	----

PF01476	1y7m	348
PF03466	1al3	2717
PF02401	----	----
PF03816	----	----
PF04397	----	----
PF07687	1cg2	1717
PF01661	1hjz	347
PF02545	1ex2	290
PF01274	1d8c	117
PF00390	1do8	114
PF03949	1do8	309
PF00629	2c9a	371
PF08125	1lj8	205
PF01050	----	----
PF01575	1iq6	27
PF01914	----	----
PF01047	1p4x	4471
PF01554	----	----
PF00917	1lb4	629
PF01824	----	----
PF03819	1vmg	114
PF03062	----	----
PF02470	----	----
PF00493	----	----
PF00015	1qu7	3635
PF03547	----	----
PF00376	1exi	2460
PF09278	1q08	323
PF01676	1ejj	241
PF00149	1aui	5179
PF01717	1ypx	467
PF01420	1yf2	583
PF04452	1vhk	94
PF08241	1f38	6399
PF08242	1bhj	1910
PF00891	1fp1	227
PF01596	1h1d	469
PF02390	1yzh	40
PF07690	1pw4	17376
PF01078	1g8p	121
PF02142	1a9x	214
PF01769	----	----
PF00129	1a1m	695
PF06623	----	----
PF00969	1k8i	7462
PF00230	2evu	814
PF00153	1okc	5843
PF03176	----	----
PF01926	1ega	3201
PF06463	1tv8	18
PF00994	1eav	1650
PF03454	1t3e	195
PF03453	1t3e	584
PF05237	1zfn	43

PF04879	1aa6	191
PF01568	1aa6	1692
PF02493	1h3i	5742
PF07661	----	----
PF03473	1oru	620
PF01618	----	----
PF01398	----	----
PF01188	1bqg	103
PF02746	1bqg	366
PF02381	1n0e	325
PF06723	1jce	409
PF00924	----	----
PF00985	1ldv	237
PF02349	----	----
PF00896	1a9o	436
PF02219	1v93	312
PF05175	1dus	546
PF02416	----	----
PF06458	----	----
PF01456	----	----
PF00893	----	----
PF01225	1cc9	1213
PF02875	1cc9	783
PF08245	1cc9	2800
PF02873	1hsk	238
PF08676	1x9z	542
PF01624	1e3m	245
PF05188	1e3m	92
PF05192	1e3m	538
PF05190	1e3m	245
PF00488	1e3m	422
PF00249	1ign	4601
PF02384	2ar0	660
PF01555	1boo	705
PF07963	1ay2	1322
PF01235	----	----
PF01699	----	----
PF03553	----	----
PF02690	----	----
PF00939	----	----
PF00175	1a8p	3335
PF03446	1pgj	2027
PF03447	1ebf	198
PF07993	----	----
PF07479	1evy	501
PF01210	1bg6	872
PF01513	1u0r	103
PF02540	1wxe	103
PF06455	----	----
PF06444	----	----
PF04095	1vlp	439
PF00931	1z6t	4504
PF00334	1b4s	599
PF00880	----	----

PF02931	1i9b	586
PF01957	----	----
PF00243	1b8k	415
PF01436	1q7f	1891
PF01292	----	----
PF03031	1t9z	126
PF00374	1cc1	207
PF01106	1veh	113
PF01592	1q48	416
PF01077	1aop	3018
PF03460	1aop	2164
PF00881	1ds7	131
PF00877	----	----
PF05368	1k6j	936
PF09084	1us4	912
PF01189	1ixk	742
PF03060	----	----
PF07391	----	----
PF01566	----	----
PF01909	1f5a	1508
PF00483	1fxo	2468
PF00293	1f3y	3719
PF08529	1hh2	334
PF01029	1eyv	643
PF02357	1m1g	514
PF08206	----	----
PF03373	----	----
PF02321	1ek9	4071
PF01276	1c4k	92
PF08352	----	----
PF00691	1oap	166
PF00215	1dbt	290
PF04069	1sw1	736
PF02784	1d7k	1205
PF00278	1d7k	294
PF00213	1abv	370
PF02566	1vla	1056
PF03968	----	----
PF00185	1a1s	100
PF02729	1a1s	958
PF00724	1bwk	516
PF00174	1ogp	107
PF00361	----	----
PF01010	----	----
PF00662	----	----
PF00420	----	----
PF00499	----	----
PF00507	----	----
PF01059	----	----
PF01058	1cc1	1109
PF00543	1gnk	575
PF00067	1akd	3730
PF02225	1cx8	533
PF05488	----	----

PF03551	1yg2	922
PF00221	1b8f	197
PF00291	1a50	2999
PF00024	1gmn	31
PF08276	----	----
PF02569	1iho	66
PF02548	1oy0	297
PF01569	1d2t	183
PF01507	1sur	450
PF01508	----	----
PF00973	1ce6	1819
PF02195	1vk1	1228
PF00740	1z1c	190
PF00989	1d06	661
PF08446	1ztu	56
PF08447	1x0o	4328
PF08448	----	----
PF03793	1k25	1031
PF01734	1oxw	1378
PF00292	1k78	216
PF01161	1a44	407
PF07943	1xp4	421
PF03717	1k25	1190
PF01395	1dqe	65
PF01851	----	----
PF01135	1i1n	480
PF03462	1zbt	327
PF07676	1c5k	2920
PF00233	1ro9	113
PF02153	----	----
PF00800	----	----
PF00595	1b8q	4468
PF00805	2bm4	1963
PF01469	----	----
PF05524	1eza	155
PF00391	1dik	1116
PF02896	1dik	1062
PF01327	1bs4	119
PF00821	1khb	604
PF03413	----	----
PF03929	----	----
PF01195	1ryb	223
PF01478	----	----
PF01252	----	----
PF00112	1aec	495
PF00548	1cqq	974
PF00863	1lvb	11
PF02902	1euv	413
PF00851	----	----
PF01433	1gw6	733
PF00413	1a85	162
PF00246	1arl	165
PF00675	1be3	624
PF05193	1be3	1116

PF00883	1gyt	37
PF02789	1gyt	173
PF01546	1cg2	1459
PF00814	1okj	155
PF01551	1qwy	1233
PF00557	1a16	1037
PF04389	1amp	524
PF01432	1i1i	833
PF01434	1ixz	856
PF01435	----	----
PF02163	----	----
PF00450	1ac5	253
PF00768	1es2	846
PF00717	1b12	459
PF02907	1a1r	2026
PF01577	----	----
PF03572	1fc6	549
PF01343	----	----
PF00949	1bef	371
PF00082	1a2q	132
PF00326	1e5t	1056
PF01136	1lk8	161
PF00532	1abe	603
PF01497	1efd	847
PF00141	1a20	1467
PF00365	1kzh	858
PF00294	1bx4	3022
PF01471	1ck7	1547
PF00300	1bif	1507
PF00342	1b0z	1253
PF00162	13pk	448
PF02878	1c47	2122
PF02879	1c47	1942
PF02880	1c47	1542
PF00408	1c47	1500
PF00169	1awe	3036
PF07167	----	----
PF02899	1a0p	199
PF00589	1crx	4096
PF00628	1wev	1231
PF02604	2a6q	53
PF02912	1eiy	312
PF02332	1fyz	20
PF01384	----	----
PF02562	----	----
PF08543	1jxh	51
PF01663	1ei6	109
PF00343	1a8i	88
PF00124	1vrn	266
PF01895	1sum	580
PF02811	1m65	1556
PF05721	2a1x	179
PF00360	----	----
PF00388	1aod	426

PF00454	1e7u	398
PF00947	2hrv	873
PF02585	1q74	250
PF07238	1ywu	401
PF01850	1w8i	1149
PF02678	1tq5	599
PF00224	1a3w	128
PF02887	1a3w	780
PF00801	1wgo	1364
PF00433	1apm	876
PF05016	1wmi	680
PF00614	----	----
PF00681	1lm5	37
PF07715	1fep	2266
PF01523	1vpb	789
PF01625	1ff3	123
PF02366	----	----
PF02478	----	----
PF01048	1a69	215
PF03726	1whu	553
PF02563	----	----
PF01743	1vfg	57
PF03364	1t17	659
PF06431	1n25	1441
PF00348	1fps	113
PF01522	1ny1	895
PF01943	----	----
PF02719	----	----
PF01558	1b0p	657
PF01855	1b0p	516
PF00267	1iiv	2014
PF01379	1ah5	85
PF03900	1ah5	184
PF00767	----	----
PF08440	----	----
PF00550	1acp	117
PF00481	1a6q	362
PF02503	1xdo	44
PF04151	1nqd	592
PF01326	1dik	709
PF01535	----	----
PF01239	1d8d	88
PF02541	1t6c	67
PF04193	----	----
PF01011	1flg	62
PF01502	1zps	331
PF01503	1yvw	205
PF00697	1dl3	133
PF05239	1eys	779
PF00874	1h99	769
PF00432	1d8d	1904
PF01255	1f75	107
PF00156	1a3c	2241
PF03991	1oei	480

PF00485	1a7j	1111
PF06325	1g6q	328
PF00484	1ddz	357
PF00160	1a33	686
PF00227	1fnt	902
PF02666	----	----
PF00223	1jb0	931
PF02468	----	----
PF01405	1s5l	596
PF01416	1dj0	132
PF00849	1prz	602
PF01437	1olz	365
PF00421	1s5l	13
PF01515	1qzt	191
PF00809	1ad1	242
PF00854	----	----
PF00381	1cm2	415
PF00358	1ax3	37
PF00359	1xiz	36
PF00367	1iba	771
PF02378	----	----
PF02302	1e2b	672
PF01472	1iq8	731
PF00806	1ib2	41
PF00787	1ocs	282
PF00070	1aog	5239
PF07992	1aog	5575
PF01243	1axj	1241
PF00282	1js3	963
PF00719	117e	674
PF01729	1o4u	455
PF02749	1o4u	481
PF00788	1ef5	533
PF08423	1szp	126
PF04002	----	----
PF04055	1olt	3997
PF03089	2jwo	1215
PF00071	121p	3167
PF00617	1bkd	270
PF02033	1jos	274
PF00415	1a12	1341
PF06271	----	----
PF00978	----	----
PF00998	1c2p	1843
PF00154	1ubc	91
PF01030	1n8y	460
PF07508	----	----
PF02132	1vdd	1
PF08534	1ewx	960
PF07494	----	----
PF04607	2be3	797
PF01421	1atl	323
PF04851	1c4o	1570
PF00239	1gdt	565

PF00072	1a2o	14128
PF03732	----	----
PF00472	1rq0	747
PF00615	1agr	87
PF01402	1q5v	18
PF07498	----	----
PF07497	----	----
PF00581	1boh	2653
PF00620	1am4	543
PF00621	1by1	1120
PF01694	----	----
PF05593	----	----
PF00073	1fod	9864
PF06026	1xtz	454
PF01872	2b3z	110
PF01042	1j7h	143
PF02867	1pem	512
PF00317	1pem	707
PF00268	1av8	212
PF00636	1u61	1033
PF03631	----	----
PF00445	1bk7	261
PF00687	1mzp	649
PF00466	1zav	74
PF00298	1aci	335
PF03946	2aw4	224
PF00542	1dd3	99
PF00572	1gs2	467
PF00238	1gs2	458
PF00252	1vqo	1457
PF01196	2cqm	606
PF01245	2aw4	35
PF00181	1yjn	1059
PF00829	----	----
PF00237	1bxe	107
PF00276	1n88	123
PF01016	2aw4	116
PF00830	----	----
PF00831	1jj2	582
PF03947	1c04	218
PF00297	1jj2	644
PF00327	1bxy	108
PF01197	2aw4	205
PF00471	2aw4	223
PF00468	2aw4	94
PF01632	2aw4	258
PF00444	2aw4	333
PF00573	1jj2	651
PF00281	1iq4	143
PF00673	1jj2	681
PF00347	1c04	295
PF01248	1ra4	19
PF03948	2aw4	7
PF01281	1cqu	419

PF00338	1fjg	422
PF00411	1fjg	657
PF00164	1fjg	958
PF00416	1fjg	92
PF00253	1fjg	45
PF00312	1a32	215
PF00886	1emw	23
PF00366	1fjg	362
PF01084	1fjg	479
PF00203	1fjg	211
PF00318	1fjg	680
PF01649	1fjg	292
PF01165	2avy	112
PF02482	1imu	425
PF00189	1fjg	470
PF00417	1fjg	411
PF00333	1fjg	492
PF03719	1fjg	551
PF01250	1cqm	534
PF00177	1fjg	896
PF00410	1an7	194
PF00380	1fjg	317
PF00834	1h1y	535
PF00652	1abr	146
PF05433	----	----
PF00355	1bcc	2849
PF00848	1eg9	600
PF04321	1kbz	169
PF07521	----	----
PF00910	----	----
PF01000	1i3q	1326
PF03118	1lb2	247
PF01193	1i3q	1472
PF04997	1i3q	418
PF00623	1i3q	1216
PF04983	1iw7	1780
PF05000	1i3q	1178
PF04998	1i3q	1721
PF04992	1i3q	296
PF05001	----	----
PF04563	1i3q	1933
PF04561	1i3q	1597
PF04565	1iw7	2260
PF04566	1i3q	556
PF04567	1i3q	1251
PF04560	1i6h	1024
PF01192	1i3q	67
PF01351	1eke	666
PF01138	2ba0	18
PF03725	1e3h	637
PF00075	1bqm	1798
PF00773	----	----
PF02508	----	----
PF00480	1woq	23

PF00639	1eq3	493
PF03055	2biw	497
PF01765	1dd5	459
PF02082	1ylf	924
PF00076	1x5s	1693
PF00398	1i4w	986
PF07634	----	----
PF00016	1aa1	14888
PF02788	1aa1	11202
PF00101	1aa1	254
PF00301	1b13	213
PF02915	1vjx	175
PF07499	1bvs	451
PF01330	1bvs	425
PF05491	1hqc	17
PF05496	1hqc	105
PF00665	1a5v	7246
PF00077	1a30	1859
PF08284	----	----
PF00078	1bqm	2879
PF07727	----	----
PF06815	1bqm	976
PF06817	1bqm	1987
PF02773	1fug	465
PF02772	1fug	537
PF00438	1fug	531
PF02574	1lt7	637
PF00575	1e3p	836
PF01479	1c05	5737
PF00954	----	----
PF03435	1e5l	335
PF08666	1vli	1
PF01259	1a48	620
PF00536	1pk1	837
PF07647	1b0x	413
PF02037	1h1j	180
PF03489	1m12	95
PF01758	----	----
PF00497	1laf	56
PF00496	1vr5	1576
PF03480	----	----
PF01297	1xvl	142
PF00188	1qnx	397
PF02036	1c44	281
PF00375	1xfh	17
PF01127	1nek	270
PF02810	1ozb	11
PF07549	----	----
PF07517	1m6n	447
PF01043	1m6n	425
PF07516	1nkt	342
PF02355	----	----
PF00584	----	----
PF03840	----	----

PF00263	----	----
PF03958	----	----
PF00344	1rh5	145
PF08238	1klx	130
PF01641	1l1d	600
PF01118	1brm	1465
PF02774	1brm	696
PF00079	1a7c	922
PF02403	1ser	84
PF00856	1h3i	1047
PF08450	1e1a	145
PF00017	1a07	315
PF07653	1hjd	571
PF08239	----	----
PF01488	1b29	1337
PF08501	1npd	729
PF01549	1bgk	106
PF00464	1bj4	781
PF04546	1sig	318
PF00140	1iw7	338
PF04539	1iw7	552
PF04545	1iw7	584
PF08281	----	----
PF03145	2a25	542
PF02146	1q14	671
PF01206	1dcj	250
PF01380	1j5x	1266
PF01202	1e6c	200
PF00395	----	----
PF01464	153l	1990
PF06470	1gxj	153
PF02481	----	----
PF01668	1k8h	140
PF01713	----	----
PF05739	3c98	37
PF09335	----	----
PF00565	1a2t	308
PF00209	2a65	345
PF00176	1z3i	1757
PF03357	----	----
PF07366	1sjw	406
PF00080	1azv	259
PF02777	1ues	905
PF00081	1d5n	1544
PF00435	1hci	3455
PF01564	1xj5	165
PF01052	1o9y	393
PF07228	----	----
PF05036	1uta	483
PF00588	1x7o	1581
PF08032	1gz0	98
PF04014	1z0r	236
PF00622	2afj	481
PF00494	----	----

PF00530	1by2	341
PF00319	1c7u	1855
PF00448	1zu4	584
PF02881	1ffh	468
PF02978	1mfq	631
PF00436	1s3o	538
PF01852	1em2	266
PF01740	1auz	449
PF07660	2d1u	545
PF01300	1hru	775
PF05922	1scj	610
PF02910	1chu	50
PF00083	1lvi	1806
PF00884	1auk	254
PF00916	----	----
PF00685	1aqu	320
PF07244	----	----
PF00084	1c1z	3345
PF04434	----	----
PF05296	----	----
PF00902	----	----
PF01026	1xwy	802
PF02668	1drt	156
PF02959	1ao7	189
PF00566	1fkm	909
PF00352	1c9b	406
PF03634	----	----
PF03741	----	----
PF00440	1sgm	6170
PF00382	1ais	32
PF00019	1nys	170
PF00688	----	----
PF02824	1nyq	1046
PF01702	1efz	37
PF00763	1a4i	610
PF02882	1a4i	416
PF00899	1r4m	1146
PF00975	1jmk	410
PF02803	1afw	1821
PF00108	1afw	1900
PF00085	1aiu	1287
PF02597	1nvi	555
PF00585	1tdj	148
PF02926	1vbk	282
PF00303	1aiq	160
PF02223	1g3u	595
PF01833	1a02	1155
PF01826	1ata	5
PF00121	1ag1	69
PF01582	1t3g	15
PF02581	1g4e	293
PF00020	1bzi	40
PF03459	1b9m	357
PF08402	1v43	417

PF03544	1u07	33
PF00593	1fep	890
PF01131	1cy0	437
PF01751	1cy0	413
PF08275	1dd9	207
PF00590	1cbf	1528
PF02775	1b0p	3006
PF00205	1bfd	1961
PF02776	1bfd	394
PF00515	1a17	10333
PF07719	1tjc	8074
PF07721	----	----
PF03151	----	----
PF01938	1uwv	1080
PF00486	2fez	6620
PF00923	1f05	565
PF02458	2bgh	538
PF01841	1evu	834
PF00912	2c5w	647
PF02779	1ay0	415
PF02780	1ay0	29
PF00456	1ay0	414
PF02133	----	----
PF00905	1e3u	3904
PF01609	1mm8	452
PF01610	----	----
PF01797	2a6m	122
PF01385	----	----
PF02371	----	----
PF03050	----	----
PF04195	----	----
PF07282	----	----
PF01527	----	----
PF01548	----	----
PF03461	----	----
PF05698	1hxv	520
PF05697	1oms	138
PF02080	1lnq	2132
PF02254	1id1	1748
PF02386	----	----
PF00133	1ffy	351
PF00579	1d2r	120
PF00749	1euq	1187
PF00750	1bs2	694
PF01406	1li5	67
PF00152	1asy	1142
PF00587	1adj	3093
PF01411	1riq	507
PF01409	1b70	793
PF01336	1asy	1084
PF01588	1b70	1263
PF01746	1oy5	501
PF03054	1gpm	367
PF07973	1nyq	980

PF00290	1a50	578
PF01509	1k8w	106
PF00234	1afh	519
PF00089	1a0h	1977
PF00090	1lsl	737
PF02412	1ux6	95
PF02956	----	----
PF03953	1ffx	3057
PF00264	1bt1	477
PF00627	1aip	1621
PF01040	----	----
PF01209	----	----
PF00240	1a5r	3297
PF00443	1nb8	741
PF03167	1akz	883
PF00984	1dli	567
PF03720	1dli	1058
PF03721	1dli	687
PF00201	2acv	636
PF02809	1p9c	663
PF00919	----	----
PF01027	----	----
PF01170	----	----
PF01458	1vh4	549
PF02130	1oz9	31
PF02367	1fl9	271
PF03652	1ovq	348
PF01594	----	----
PF03458	----	----
PF03692	----	----
PF00179	1a3s	321
PF01208	1j93	449
PF00582	1mjh	878
PF07702	2fa1	555
PF02151	1e52	490
PF08459	----	----
PF00580	1pjr	806
PF07686	1a14	977
PF00559	1vzf	16
PF01443	----	----
PF01660	----	----
PF00695	----	----
PF00654	1kpk	143
PF00434	----	----
PF00558	1pi7	1450
PF00092	1ao3	2858
PF00093	----	----
PF00094	----	----
PF03716	----	----
PF00400	1b9x	2155
PF00110	----	----
PF03106	1wj2	193
PF00397	1e0l	1126
PF04932	----	----

PF02706	----	----
PF00739	----	----
PF00860	----	----
PF02162	----	----
PF00102	1a5y	677
PF04073	1dbu	553
PF04264	1wub	160
PF02325	----	----
PF03853	1jzt	476
PF03739	----	----
PF04650	----	----
PF07776	1pzw	189
PF00643	2csv	690
PF00096	2gli	26754
PF00097	1bor	2590
PF00105	2gda	2181
PF06689	1ovx	232
PF01396	----	----
PF00642	1m9o	2144
PF00098	1a1t	997
PF01807	1d0q	208
PF01529	----	----
PF01258	1tjl	308
PF06827	1ffy	90
PF01753	----	----
PF01422	----	----
PF00641	1n0z	40
PF02535	----	----
PF00172	1ajy	574
PF00100	----	----
PF00069	1a06	2869
PF00005	1b0u	17573
