# Supplementary material for: Identification of Coevolving Residues and Coevolution Potentials Emphasizing Structure, Bond Formation and Catalytic Coordination in Protein Evolution
Source: PLoS One. 2009 Mar 10;4(3):e4762. doi: 10.1371/journal.pone.0004762 (PMC2651771; doi:10.1371/journal.pone.0004762)
Supplement: Table S3 — (0.03 MB PDF) [file pone.0004762.s009.pdf]

Table S3. CSA entries for analyzed catalytic sites

[PDB ID,SITE NUMBER,RESIDUE TYPE,CHAIN ID,RESIDUE NUMBER,CHEMICAL FUNCTION,EVIDENCE TYPE,LITERATURE ENTRY]
1aro,0,TYR,L,1046,S,PSIBLAST,1lbaA
1e6v,0,GLY,A,468,S,PSIBLAST,1mroA
1aos,0,HIS,A,160,S,PSIBLAST,1auwA
1aos,0,THR,A,159,S,PSIBLAST,1auwA
1aos,1,SER,A,281,S,PSIBLAST,1auwA
1aos,1,LYS,A,287,S,PSIBLAST,1auwA
1aos,1,GLU,A,294,S,PSIBLAST,1auwA
1b5s,0,ARG,A,206,S,PSIBLAST,3claA
1b5s,0,ASP,A,402,S,PSIBLAST,3claA
1b5s,0,HIS,A,398,S,PSIBLAST,3claA
1f0y,0,ASN,A,208,X,PSIBLAST,2hdhA
1f0y,0,SER,A,137,X,PSIBLAST,2hdhA
1f0y,0,HIS,A,158,X,PSIBLAST,2hdhA
1bgx,0,LYS,T,82,S,PSIBLAST,1exnA
1ho5,0,ARG,A,410,S,PSIBLAST,1ushA
1ho5,0,ARG,A,379,S,PSIBLAST,1ushA
1pgj,0,GLU,A,192,S,PSIBLAST,2pgdA
1pgj,0,LYS,A,185,S,PSIBLAST,2pgdA
1pgj,0,ASN,A,189,S,PSIBLAST,2pgdA
1a4l,0,HIS,A,238,S,LIT,1a4l
1a4l,0,TYR,A,240,S,LIT,1a4l
1a4l,0,GLU,A,217,S,LIT,1a4l
1a4l,0,ASP,A,295,S,LIT,1a4l
1b7b,0,LYS,A,209,S,PSIBLAST,1e19A
1b7b,0,LYS,A,271,S,PSIBLAST,1e19A
1b7b,0,LYS,A,128,S,PSIBLAST,1e19A
1d2n,0,LYS,A,557,S,PSIBLAST,1nsfA
1a7u,0,SER,A,98,S,LIT,1a7u
1a7u,2,SER,A,98,S,PSIBLAST,1azwA
1a7u,0,MET,A,99,N,LIT,1a7u
1auo,0,HIS,A,199,S,LIT,1auo
1auo,0,SER,A,114,S,LIT,1auo
1auo,0,GLN,A,115,N,LIT,1auo
1auo,0,LEU,A,23,N,LIT,1auo
1auo,0,ASP,A,168,S,LIT,1auo
1evq,0,SER,A,155,S,PSIBLAST,1jkmA
1evq,0,HIS,A,282,S,PSIBLAST,1jkmA
1evq,0,ASP,A,252,S,PSIBLAST,1jkmA
1g99,0,GLY,A,212,N,LIT,1g99
1g99,0,SER,A,10,S,LIT,1g99
1g99,0,ASP,A,148,S,LIT,1g99
1g99,0,ARG,A,91,S,LIT,1g99
1g99,0,ARG,A,241,S,LIT,1g99
1b6b,1,SER,A,97,S,LIT,1b6b
1b6b,1,HIS,A,122,S,LIT,1b6b
1b6b,1,LEU,A,124,N,LIT,1b6b
1b6b,1,TYR,A,168,S,LIT,1b6b

1cvi,0,ARG,A,1079,S,PSIBLAST,1rptA
1cvi,0,ARG,A,1011,S,PSIBLAST,1rptA
1cvi,0,HIS,A,1257,S,PSIBLAST,1rptA
1cvi,0,HIS,A,1012,S,PSIBLAST,1rptA
1cvi,0,ASP,A,1258,S,PSIBLAST,1rptA
1cvi,0,ARG,A,1015,S,PSIBLAST,1rptA
1ebl,0,CYS,A,112,S,PSIBLAST,1cgkA
1ebl,0,PHE,A,157,S,PSIBLAST,1cgkA
1ebl,0,ASN,A,274,S,PSIBLAST,1cgkA
1ebl,0,HIS,A,244,S,PSIBLAST,1cgkA
1f7l,1,LYS,A,62,X,PSIBLAST,1f80C
1f7l,2,LYS,A,62,S,LIT,1f7l
1buc,0,GLY,A,246,S,PSIBLAST,1ivhA
1buc,2,GLU,A,367,S,PSIBLAST,3mddA
1gxt,0,ARG,A,23,S,PSIBLAST,2acyA
1gxt,0,ASN,A,41,S,PSIBLAST,2acyA
1iuq,0,HIS,A,139,S,PSIBLAST,1k30A
1iuq,0,ASP,A,144,S,PSIBLAST,1k30A
1ade,0,ASP,A,13,S,PSIBLAST,1gimA
1ade,0,GLN,A,224,S,PSIBLAST,1gimA
1ade,0,HIS,A,41,S,PSIBLAST,1gimA
1a71,0,LEU,A,57,S,PSIBLAST,1gufA
1a71,2,SER,A,48,S,PSIBLAST,1qlhA
1a71,2,HIS,A,51,S,PSIBLAST,1qlhA
1a7a,0,CYS,A,195,S,PSIBLAST,1b3rA
1a7a,0,LYS,A,186,S,PSIBLAST,1b3rA
1a7a,0,ASP,A,131,S,PSIBLAST,1b3rA
1a7a,0,ASN,A,191,S,PSIBLAST,1b3rA
1a7a,0,HIS,A,55,S,PSIBLAST,1b3rA
1a7a,0,HIS,A,301,S,PSIBLAST,1b3rA
1a7a,0,CYS,A,195,S,PSIBLAST,1b3rA
1a7a,0,ASN,A,191,S,PSIBLAST,1b3rA
1a7a,0,HIS,A,301,S,PSIBLAST,1b3rA
1g8m,0,ASN,A,432,S,PSIBLAST,1p4rA
1g8m,0,HIS,A,268,S,PSIBLAST,1p4rA
1g8m,0,LYS,A,267,S,PSIBLAST,1p4rA
1g8m,2,LYS,A,138,S,PSIBLAST,1p4rA
1bd0,1,CYS,A,311,S,LIT,1bd0
1pjb,0,ASP,A,269,S,LIT,1pjb
1pjb,0,GLU,A,117,S,LIT,1pjb
1pjb,0,LYS,A,74,S,LIT,1pjb
1pjb,0,HIS,A,95,S,LIT,1pjb
1a4s,0,ASN,A,166,N,LIT,1a4s
1a4s,0,CYS,A,297,S,LIT,1a4s
1a4s,0,GLU,A,263,S,LIT,1a4s
1dzu,0,TYR,P,113,S,PSIBLAST,1fuaA
1dzu,0,GLU,P,73,S,PSIBLAST,1fuaA
1dzu,1,ALA,P,117,S,PSIBLAST,1k0wA
1aja,0,SER,A,102,S,PSIBLAST,1alkA
1aja,0,ARG,A,166,S,PSIBLAST,1alkA
1m21,0,SER,A,226,S,LIT,1m21

1m21,0,LYS,A,123,S,LIT,1m21
1m21,0,SER,A,202,S,LIT,1m21
1k6w,0,GLY,A,305,S,PSIBLAST,1ybqA
1d7r,0,TRP,A,138,X,PSIBLAST,1cs1A
1d7r,1,TRP,A,138,S,LIT,1d7r
1d7r,0,ASP,A,243,X,PSIBLAST,1cs1A
1d7r,1,ASP,A,243,S,LIT,1d7r
1d7r,1,LYS,A,272,S,LIT,1d7r
1a0g,0,ALA,A,201,S,PSIBLAST,1daaA
1a0g,0,LYS,A,145,S,PSIBLAST,1daaA
1a0g,0,GLU,A,177,S,PSIBLAST,1daaA
1bjn,0,TRP,A,102,X,PSIBLAST,1bjoA
1bjn,0,ASP,A,174,X,PSIBLAST,1bjoA
1amu,0,LYS,A,434,S,PSIBLAST,1v25A
1a0c,0,HIS,A,270,S,PSIBLAST,1de6A
1a0c,0,LYS,A,233,S,PSIBLAST,1de6A
1a0c,4,LYS,A,233,S,PSIBLAST,2xisA
1a0c,4,GLU,A,231,S,PSIBLAST,2xisA
1j7i,0,LYS,A,44,S,PSIBLAST,1l8tA
1j7i,0,ASP,A,190,S,PSIBLAST,1l8tA
1e0s,0,GLN,A,67,S,PSIBLAST,1ksjA
1cev,0,ASP,A,126,S,LIT,1cev
1cev,0,GLU,A,271,S,LIT,1cev
1j1z,0,ASP,A,121,S,PSIBLAST,1kp2A
1j1z,0,ARG,A,92,S,PSIBLAST,1kp2A
1ct9,0,HIS,A,430,X,PSIBLAST,1jxaC
1b73,0,CYS,A,70,S,LIT,1b73
1b73,1,CYS,A,70,S,PSIBLAST,1jflA
1euc,1,GLU,B,204,X,PSIBLAST,1cqjB
1bmf,6,LYS,A,175,X,PSIBLAST,1ohhE
1bmf,6,LYS,A,209,X,PSIBLAST,1ohhE
1bmf,6,GLN,A,208,X,PSIBLAST,1ohhE
1r30,0,ARG,A,260,S,LIT,1r30
1ax4,0,LLP,A,266,S,LIT,1ax4
1ax4,0,ASP,A,223,S,LIT,1ax4
1ax4,4,ASP,A,223,S,PSIBLAST,1d7rA
1ax4,8,ASP,A,223,S,PSIBLAST,1elqA
1ax4,12,ASP,A,223,S,PSIBLAST,1qz9A
1ax4,0,PHE,A,132,S,LIT,1ax4
1ax4,12,PHE,A,132,S,PSIBLAST,1qz9A
1ax4,4,LYS,A,265,S,PSIBLAST,1d7rA
1ax4,4,PHE,A,130,S,PSIBLAST,1d7rA
1ax4,8,HIS,A,131,S,PSIBLAST,1elqA
1ax4,8,LYS,A,255,S,PSIBLAST,1elqA
1ax4,12,LYS,A,255,S,PSIBLAST,1qz9A
1ax4,16,ARG,A,76,X,PSIBLAST,1cs1C
1kyh,0,LYS,A,186,S,PSIBLAST,1jxhA
1kyh,0,GLY,A,213,N,PSIBLAST,1jxhA
1kyh,1,GLY,A,213,N,PSIBLAST,1tz3A
1kyh,1,GLY,A,215,N,PSIBLAST,1tz3A
1kyh,1,ASP,A,216,SN,PSIBLAST,1tz3A

1kyh,1,THR,A,214,N,PSIBLAST,1tz3A
1a4e,0,SER,A,109,S,PSIBLAST,1iphA
1a4e,0,ASN,A,143,S,PSIBLAST,1iphA
1a4e,0,HIS,A,70,S,PSIBLAST,1iphA
1bi5,0,ASN,A,336,X,PSIBLAST,1cgkA
1bi5,0,HIS,A,303,X,PSIBLAST,1cgkA
1bi5,0,PHE,A,215,X,PSIBLAST,1cgkA
1a2o,0,ASP,A,286,S,PSIBLAST,1chdA
1a2o,0,SER,A,164,S,PSIBLAST,1chdA
1a2o,0,MET,A,283,N,PSIBLAST,1chdA
1a2o,0,THR,A,165,N,PSIBLAST,1chdA
1a2o,0,HIS,A,190,S,PSIBLAST,1chdA
1i1q,0,HIS,A,398,X,PSIBLAST,1i7qA
1csm,0,ARG,A,16,S,PSIBLAST,3csmA
1kp9,0,GLU,A,140,S,PSIBLAST,1kywA
1p5h,0,ASP,A,169,S,PSIBLAST,1xvtA
1p5h,3,ASP,A,169,S,PSIBLAST,1t4cA
1p5h,2,GLY,A,260,N,PSIBLAST,1t4cA
1p5h,2,GLY,A,261,N,PSIBLAST,1t4cA
1p5h,3,GLU,A,140,N,PSIBLAST,1t4cA
1a65,0,CYS,A,452,SO,LIT,1a65
1a65,0,HIS,A,453,SNO,LIT,1a65
1a65,0,HIS,A,451,S,LIT,1a65
1o4t,0,GLU,A,68,S,PSIBLAST,1gqgA
1cl1,0,TYR,A,111,S,LIT,1cl1
1cl1,0,ASP,A,185,S,LIT,1cl1
1cl1,0,LLP,A,210,S,LIT,1cl1
1cl1,3,ARG,A,58,X,PSIBLAST,1cs1A
1aof,0,HIS,A,388,S,PSIBLAST,1nirA
1aof,0,HIS,A,345,S,PSIBLAST,1nirA
1d9e,0,HIS,A,1202,S,PSIBLAST,1q3nA
1bwz,0,CYS,A,73,S,LIT,1bwz
1arz,0,HIS,A,159,S,LIT,1arz
1arz,0,LYS,A,163,S,LIT,1arz
1af2,0,GLU,A,104,S,PSIBLAST,1cttA
1b9h,0,PHE,A,88,X,PSIBLAST,1cs1A
1b9h,2,PHE,A,88,S,LIT,1b9h
1b9h,1,ARG,A,34,X,PSIBLAST,1cs1C
1b9h,2,LYS,A,188,S,LIT,1b9h
1j2w,0,LYS,A,180,S,PSIBLAST,1p1xA
1j2w,0,LYS,A,151,S,PSIBLAST,1p1xA
1j2w,0,ASP,A,89,S,PSIBLAST,1p1xA
1p9o,0,ASN,A,59,S,PSIBLAST,1u7uA
1dhp,0,ARG,A,138,S,LIT,1dhp
1dhp,0,TYR,A,133,S,LIT,1dhp
1dhp,0,LYS,A,161,S,LIT,1dhp
1dhp,2,LYS,A,161,S,PSIBLAST,1fdyA
1dhp,2,THR,A,44,N,PSIBLAST,1fdyA
1dhp,2,THR,A,45,N,PSIBLAST,1fdyA
1ai9,0,VAL,A,10,O,PSIBLAST,1ra2A
1ai9,0,GLU,A,32,S,PSIBLAST,1ra2A

1ai9,0,ILE,A,33,S,PSIBLAST,1ra2A
1ai9,0,MET,A,25,S,PSIBLAST,1ra2A
1ai9,0,ILE,A,112,O,PSIBLAST,1ra2A
1ai9,0,LEU,A,69,S,PSIBLAST,1ra2A
1ai9,0,PHE,A,36,S,PSIBLAST,1ra2A
1d3g,0,SER,A,215,S,PSIBLAST,1h7xA
1d3g,1,SER,A,215,S,LIT,1d3g
1d3g,1,THR,A,218,S,LIT,1d3g
1d3g,1,PHE,A,149,S,LIT,1d3g
1d3g,1,LYS,A,255,S,LIT,1d3g
1dqs,0,HIS,A,275,S,LIT,1dqs
1dlm,0,ARG,A,221,S,PSIBLAST,3pcaM
1dlm,0,TYR,A,200,S,PSIBLAST,3pcaM
1c2y,0,HIS,A,88,S,PSIBLAST,1rvvA
1eh6,0,GLU,A,172,S,LIT,1eh6
1eh6,0,CYS,A,145,S,LIT,1eh6
1eh6,0,ASN,A,137,S,LIT,1eh6
1eh6,0,HIS,A,146,S,LIT,1eh6
1b04,0,ALA,A,114,S,LIT,1b04
1d5r,0,ARG,A,130,S,LIT,1d5r
1d5r,1,ARG,A,130,S,PSIBLAST,1pa9A
1d5r,0,CYS,A,124,S,LIT,1d5r
1d5r,1,CYS,A,124,S,PSIBLAST,1pa9A
1d5r,1,THR,A,131,S,PSIBLAST,1pa9A
1dzr,0,ASP,A,170,S,LIT,1dzr
1dzr,0,HIS,A,63,S,LIT,1dzr
1vhn,0,CYS,A,93,S,PSIBLAST,1h7xA
1dtw,0,HIS,A,291,S,LIT,1dtw
1dtw,1,HIS,A,291,S,LIT,1dtw
1dci,0,ASP,A,204,S,LIT,1dci
1dci,0,GLU,A,196,S,LIT,1dci
1dci,3,GLU,A,196,S,PSIBLAST,1hzdA
1dci,3,GLY,A,173,N,PSIBLAST,1hzdA
1dci,3,ASP,A,176,S,PSIBLAST,1hzdA
1e9i,0,GLU,A,208,S,PSIBLAST,1elsA
1e9i,4,GLU,A,208,S,PSIBLAST,5enlA
1e9i,0,GLU,A,167,S,PSIBLAST,1elsA
1e9i,4,GLU,A,167,S,PSIBLAST,5enlA
1e9i,8,HIS,A,191,S,PSIBLAST,1kczA
1e9i,12,LYS,A,230,S,PSIBLAST,1r6wA
1f6d,0,GLU,A,131,S,LIT,1f6d
1f6d,0,ASP,A,95,S,LIT,1f6d
1f6d,0,GLU,A,117,S,LIT,1f6d
1f6d,0,HIS,A,213,S,LIT,1f6d
1fxx,0,HIS,A,181,S,PSIBLAST,1w0hA
1fxx,0,GLU,A,17,S,PSIBLAST,1w0hA
1b57,0,ASN,A,286,S,LIT,1b57
1b57,0,GLU,A,182,S,LIT,1b57
1mka,0,CYS,A,80,N,LIT,1mka
1mka,0,VAL,A,76,O,LIT,1mka
1mka,0,GLY,A,79,N,LIT,1mka

1mka,0,HIS,A,70,S,LIT,1mka
1mka,1,ASP,A,84,S,LIT,1mka
1ahu,0,HIS,A,422,S,PSIBLAST,1vaoA
1ahu,0,TYR,A,503,S,PSIBLAST,1vaoA
1amo,0,SER,A,457,S,LIT,1amo
1amo,2,TYR,A,456,S,PSIBLAST,1ndhA
1ahu,0,ASP,A,170,S,PSIBLAST,1vaoA
1dnp,0,TRP,A,382,S,LIT,1dnp
1ee8,0,GLU,A,2,S,PSIBLAST,1k82A
1ee8,0,LYS,A,52,S,PSIBLAST,1k82A
1ee8,0,PRO,A,1,S,PSIBLAST,1k82A
1jpu,0,ASN,A,260,S,PSIBLAST,1dqsA
1jpu,1,HIS,A,256,S,PSIBLAST,1dqsA
1a7x,0,ILE,A,56,N,PSIBLAST,1d6oA
1a7x,0,TYR,A,82,S,PSIBLAST,1d6oA
1a7x,0,ASP,A,37,S,PSIBLAST,1d6oA
1d4a,0,TYR,A,155,S,LIT,1d4a
1d4a,0,GLY,A,149,N,LIT,1d4a
1e20,0,HIS,A,90,S,PSIBLAST,1mvnA
1b9l,0,LYS,A,102,S,PSIBLAST,2dhnA
1b9l,0,GLU,A,25,S,PSIBLAST,2dhnA
1c2t,0,SER,A,135,S,LIT,1c2t
1c2t,0,ASN,A,106,S,LIT,1c2t
1c2t,2,ASN,A,106,S,PSIBLAST,1cdeA
1c2t,0,ASP,A,144,S,LIT,1c2t
1c2t,2,ASP,A,144,S,PSIBLAST,1cdeA
1c2t,4,ASP,A,144,S,PSIBLAST,1s3iA
1c2t,0,HIS,A,108,S,LIT,1c2t
1c2t,2,HIS,A,108,S,PSIBLAST,1cdeA
1c2t,4,HIS,A,108,S,PSIBLAST,1s3iA
1dpg,0,HIS,A,240,S,LIT,1dpg
1dpg,0,ASP,A,177,S,LIT,1dpg
1c0a,0,ALA,A,320,S,PSIBLAST,1b7yA
1ao0,4,GLY,A,103,S,PSIBLAST,1ecfA
1ao0,4,ASN,A,102,S,PSIBLAST,1ecfA
1pj5,0,ASP,A,552,S,LIT,1pj5
1evj,0,LYS,A,129,S,PSIBLAST,1ofgA
1evj,0,TYR,A,217,S,PSIBLAST,1ofgA
1ea0,2,LYS,A,937,S,PSIBLAST,1ofdA
1ea0,2,GLU,A,886,S,PSIBLAST,1ofdA
1cd5,0,HIS,A,143,S,LIT,1cd5
1cd5,0,GLU,A,148,S,LIT,1cd5
1cd5,0,ASP,A,141,S,LIT,1cd5
1cd5,0,ASP,A,72,S,LIT,1cd5
1bga,0,GLU,A,352,S,PSIBLAST,1cbgA
1bga,4,GLU,A,352,S,PSIBLAST,1pbgA
1bga,12,GLU,A,352,S,PSIBLAST,1fhlA
1bga,0,GLU,A,166,S,PSIBLAST,1cbgA
1bga,4,GLU,A,166,S,PSIBLAST,1pbgA
1bga,8,GLU,A,166,S,PSIBLAST,1pz3A
1bga,12,GLU,A,166,S,PSIBLAST,1fhlA

1bga,16,GLU,A,166,S,PSIBLAST,1cz1A
1bga,0,ASN,A,294,S,PSIBLAST,1cbgA
1bga,12,ARG,A,77,S,PSIBLAST,1fhlA
1bga,16,ASP,A,265,S,PSIBLAST,1cz1A
1b30,0,GLU,A,132,S,PSIBLAST,1expA
1b30,1,GLU,A,132,S,PSIBLAST,2hisA
1aq0,0,GLU,A,280,S,LIT,1aq0
1aq0,0,LYS,A,283,S,LIT,1aq0
1aq0,0,GLU,A,288,S,LIT,1aq0
1aq0,2,GLU,A,288,S,PSIBLAST,1ghsB
1aq0,0,GLU,A,232,S,LIT,1aq0
1aq0,2,GLU,A,232,S,PSIBLAST,1ghsB
1c3f,0,ASN,A,130,S,PSIBLAST,2ebnA
1c3f,0,GLU,A,132,S,PSIBLAST,2ebnA
1cns,0,SER,A,120,S,LIT,1cns
1xsi,0,ASP,A,482,S,PSIBLAST,1uasA
1xsi,0,ASP,A,416,S,PSIBLAST,1uasA
1st8,0,GLU,A,201,S,PSIBLAST,1y9mA
1g9r,0,GLN,A,189,S,PSIBLAST,1ga8A
1g9r,0,ASN,A,153,S,PSIBLAST,1ga8A
1azy,0,HIS,A,85,S,LIT,1azy
1azy,2,HIS,A,85,S,PSIBLAST,1brwA
1azy,0,ARG,A,171,S,LIT,1azy
1azy,2,ARG,A,171,S,PSIBLAST,1brwA
1azy,0,LYS,A,190,S,LIT,1azy
1azy,2,LYS,A,190,S,PSIBLAST,1brwA
1bh5,0,GLN,A,172,S,PSIBLAST,1froA
1cf3,0,HIS,A,516,S,PSIBLAST,1galA
1cf3,1,HIS,A,516,S,PSIBLAST,1kdgA
1cf3,0,HIS,A,559,S,PSIBLAST,1galA
1cf3,1,HIS,A,559,S,PSIBLAST,1kdgA
1a7k,0,HIS,A,194,S,PSIBLAST,1szjG
1a7k,0,CYS,A,166,S,PSIBLAST,1szjG
1gp1,0,GLN,A,80,S,LIT,1gp1
1gp1,0,SEC,A,45,S,LIT,1gp1
1a8r,0,HIS,A,179,S,PSIBLAST,1gtpA
1a8r,0,SER,A,112,S,PSIBLAST,1gtpA
1aip,4,HIS,A,85,S,PSIBLAST,1ksjA
1ab8,0,ARG,A,1029,S,LIT,1ab8
1c4z,0,CYS,A,820,S,LIT,1c4z
1c4z,0,HIS,A,818,S,LIT,1c4z
1c4z,0,ASP,A,607,S,LIT,1c4z
1diz,0,ASP,A,238,S,LIT,1diz
1diz,0,TYR,A,222,S,LIT,1diz
1gpw,6,ASN,A,11,S,PSIBLAST,2a0nA
1gpw,6,ASP,A,130,S,PSIBLAST,2a0nA
1k75,0,GLU,A,326,S,PSIBLAST,1kaeA
1k75,0,HIS,A,327,S,PSIBLAST,1kaeA
1av5,0,HIS,A,112,S,PSIBLAST,5fitA
1av5,0,ASN,A,99,S,PSIBLAST,5fitA
1av5,0,HIS,A,110,S,PSIBLAST,5fitA

1nvm,0,HIS,A,21,S,LIT,1nvm
1bxi,0,HIS,B,103,S,PSIBLAST,1znvB
1bxi,1,HIS,B,103,S,PSIBLAST,1fr2B
1bxi,1,HIS,B,102,S,PSIBLAST,1fr2B
1bxi,1,GLU,B,100,S,PSIBLAST,1fr2B
1ebf,0,LYS,A,223,S,LIT,1ebf
1ebf,0,ASP,A,219,S,LIT,1ebf
1dxe,0,ARG,A,75,S,LIT,1dxe
1dxe,0,HIS,A,50,S,LIT,1dxe
1cbk,0,ARG,A,83,S,PSIBLAST,1hkaA
1aq6,0,GLY,A,116,S,PSIBLAST,1lvhA
1aq6,2,ASP,A,8,S,PSIBLAST,1qh9A
1aq6,4,ASP,A,8,S,PSIBLAST,1qq5A
1aq6,2,SER,A,114,S,PSIBLAST,1qh9A
1aq6,2,ARG,A,39,S,PSIBLAST,1qh9A
1aq6,4,ARG,A,39,S,PSIBLAST,1qq5A
1aq6,2,ASP,A,176,S,PSIBLAST,1qh9A
1aq6,4,ASP,A,176,S,PSIBLAST,1qq5A
1aq6,4,THR,A,12,S,PSIBLAST,1qq5A
1aq6,4,PHE,A,175,S,PSIBLAST,1qq5A
1aq6,4,ASN,A,173,S,PSIBLAST,1qq5A
1aq6,4,ASN,A,115,S,PSIBLAST,1qq5A
1aq6,4,SER,A,171,S,PSIBLAST,1qq5A
1dqu,0,HIS,A,195,X,PSIBLAST,1f8mA
1np3,0,GLU,A,230,S,PSIBLAST,1yveI
1awb,0,THR,A,95,S,PSIBLAST,1imaA
1awb,0,GLU,A,70,S,PSIBLAST,1imaA
1a05,0,TYR,A,140,S,LIT,1a05
1a05,1,ASP,A,222,S,LIT,1a05
1a05,1,LYS,A,190,S,LIT,1a05
1ilw,0,ASP,A,10,S,PSIBLAST,1nf9A
1ilw,1,ASP,A,10,S,PSIBLAST,1im5A
1ilw,1,CYS,A,133,SN,PSIBLAST,1im5A
1ilw,1,ALA,A,129,N,PSIBLAST,1im5A
1ilw,1,LYS,A,94,S,PSIBLAST,1im5A
1fwy,0,ARG,A,18,S,PSIBLAST,1hv9A
1ezr,0,ASP,A,10,S,PSIBLAST,1masA
1ezr,0,HIS,A,240,S,PSIBLAST,1masA
1ezr,0,ASN,A,168,S,PSIBLAST,1masA
1b3n,0,CYS,A,163,S,PSIBLAST,1kasA
1b3n,1,CYS,A,163,SN,PSIBLAST,1dd8A
1b3n,0,HIS,A,340,S,PSIBLAST,1kasA
1b3n,1,HIS,A,340,S,PSIBLAST,1dd8A
1b3n,0,HIS,A,303,S,PSIBLAST,1kasA
1b3n,1,HIS,A,303,SO,PSIBLAST,1dd8A
1b3n,1,LYS,A,335,S,PSIBLAST,1dd8A
1nn4,0,HIS,A,10,S,LIT,1nn4
1nn4,0,ARG,A,137,S,LIT,1nn4
1nn4,0,CYS,A,66,S,LIT,1nn4
1nn4,0,ASP,A,9,S,LIT,1nn4
1a7t,0,ASP,A,86,S,PSIBLAST,2bmiA

1a7t,2,ASP,A,86,S,PSIBLAST,1qh5A
1a7t,0,ASN,A,176,S,PSIBLAST,2bmiA
1a7t,4,GLN,A,170,S,PSIBLAST,1smlA
1kzl,0,ASP,A,185,S,LIT,1kzl
1kzl,0,SER,A,146,S,LIT,1kzl
1kzl,1,HIS,A,102,S,PSIBLAST,1i8dA
1d8c,0,ARG,A,338,S,LIT,1d8c
1do8,0,ASP,A,278,S,LIT,1do8
1do8,0,LYS,A,183,S,LIT,1do8
1do8,0,TYR,A,112,S,LIT,1do8
1iq6,0,ILE,A,51,O,PSIBLAST,1s9cA
1iq6,0,ASP,A,31,S,PSIBLAST,1s9cA
1iq6,0,GLY,A,54,N,PSIBLAST,1s9cA
1iq6,0,HIS,A,36,S,PSIBLAST,1s9cA
1aui,0,ASP,A,121,S,LIT,1aui
1aui,1,ASP,A,121,S,PSIBLAST,1s95A
1aui,0,HIS,A,151,S,LIT,1aui
1aui,1,HIS,A,151,S,PSIBLAST,1s95A
1aui,1,HIS,A,281,S,PSIBLAST,1s95A
1aui,1,ARG,A,122,S,PSIBLAST,1s95A
1aui,1,ASN,A,150,S,PSIBLAST,1s95A
1fp1,0,HIS,D,278,S,PSIBLAST,1kywA
1h1d,0,LYS,A,144,S,PSIBLAST,1vidA
1ega,0,LEU,A,66,S,PSIBLAST,1ksjA
1jce,0,PRO,A,64,S,PSIBLAST,1kazA
1jce,1,LYS,A,49,S,PSIBLAST,1kazA
1v93,0,ASP,A,109,S,PSIBLAST,1b5tA
1v93,0,GLU,A,18,S,PSIBLAST,1b5tA
1dus,0,ASP,A,84,S,PSIBLAST,1qamA
1dus,0,ASN,A,129,S,PSIBLAST,1qamA
1dus,0,GLY,A,63,O,PSIBLAST,1qamA
1cc9,0,LYS,A,130,S,PSIBLAST,1p3dA
1hsk,0,SER,A,238,S,PSIBLAST,1mbbA
1hsk,0,GLU,A,308,S,PSIBLAST,1mbbA
1pgj,0,GLY,A,132,N,PSIBLAST,2pgdA
1evy,0,LYS,A,210,S,LIT,1evy
1evy,0,THR,A,267,S,LIT,1evy
1b4s,0,ASN,A,119,X,PSIBLAST,1nspA
1b4s,0,LYS,A,16,X,PSIBLAST,1nspA
1b4s,3,LYS,A,16,X,PSIBLAST,1paeX
1b4s,3,TYR,A,56,X,PSIBLAST,1paeX
1fxo,0,ARG,A,15,S,PSIBLAST,1hv9A
1c4k,0,ASP,A,316,S,PSIBLAST,1ordA
1c4k,0,LYS,A,355,S,PSIBLAST,1ordA
1dbt,0,LYS,A,62,S,LIT,1dbt
1dbt,3,LYS,A,62,S,PSIBLAST,1eixA
1dbt,3,LYS,A,33,S,PSIBLAST,1eixA
1dbt,3,ASP,A,65,S,PSIBLAST,1eixA
1d7k,0,LYS,A,161,X,PSIBLAST,1bd0A
1d7k,0,LLP,A,69,X,PSIBLAST,1bd0A
1d7k,2,ARG,A,165,X,PSIBLAST,1bd0A

1d7k,4,GLU,A,274,X,PSIBLAST,7odcA
1d7k,4,HIS,A,197,X,PSIBLAST,7odcA
1bwk,0,ASN,A,191,S,PSIBLAST,1oyaA
1bwk,0,ASN,A,194,S,PSIBLAST,1oyaA
1bwk,0,TYR,A,196,S,PSIBLAST,1oyaA
1b8f,0,ALA,A,142,S,LIT,1b8f
1b8f,0,TYR,A,280,S,LIT,1b8f
1b8f,0,SER,A,143,S,LIT,1b8f
1b8f,0,GLU,A,414,S,LIT,1b8f
1b8f,0,GLY,A,144,S,LIT,1b8f
1a50,0,HIS,B,86,S,LIT,1a50
1a50,0,LYS,B,87,S,LIT,1a50
1a50,1,LYS,B,87,S,PSIBLAST,1pwhA
1a50,2,LYS,B,87,S,PSIBLAST,1tdjA
1a50,0,ASP,B,305,S,LIT,1a50
1a50,2,SER,B,377,S,PSIBLAST,1tdjA
1d2t,0,LYS,A,115,S,PSIBLAST,1vncA
1d2t,0,HIS,A,150,S,PSIBLAST,1vncA
1d2t,1,HIS,A,150,S,LIT,1d2t
1d2t,1,HIS,A,189,S,LIT,1d2t
1d2t,1,ASP,A,193,S,LIT,1d2t
1d2t,1,ARG,A,183,S,LIT,1d2t
1bs4,0,GLU,A,133,S,LIT,1bs4
1bs4,0,GLY,A,45,N,LIT,1bs4
1bs4,0,LEU,A,91,N,LIT,1bs4
1bs4,0,GLN,A,50,S,LIT,1bs4
1khb,0,ARG,A,405,S,PSIBLAST,1aq2A
1khb,1,ARG,A,405,S,PSIBLAST,1nhxA
1ryb,0,HIS,A,21,S,PSIBLAST,2pthA
1ryb,0,ASP,A,93,S,PSIBLAST,2pthA
1cqq,0,GLY,A,145,N,LIT,1cqq
1cqq,0,HIS,A,40,S,LIT,1cqq
1cqq,0,GLU,A,71,S,LIT,1cqq
1euv,0,ASP,A,531,S,PSIBLAST,2bkrA
1euv,0,HIS,A,514,S,PSIBLAST,2bkrA
1euv,0,TRP,A,515,S,PSIBLAST,2bkrA
1gw6,0,TYR,A,383,S,PSIBLAST,1h19A
1gw6,0,GLU,A,296,S,PSIBLAST,1h19A
1gw6,0,GLU,A,271,S,PSIBLAST,1h19A
1a85,0,MET,A,215,S,PSIBLAST,1hfsA
1a85,0,GLU,A,198,S,PSIBLAST,1hfsA
1a85,1,GLU,A,198,S,PSIBLAST,1qibA
1be3,1,GLU,A,60,S,PSIBLAST,1hr6B
1gyt,0,ARG,A,356,S,PSIBLAST,1lamA
1gyt,0,ASP,A,275,S,PSIBLAST,1lamA
1gyt,0,LYS,A,282,S,PSIBLAST,1lamA
1cg2,0,GLU,A,175,S,PSIBLAST,1ampA
1cg2,4,GLU,A,175,S,LIT,1cg2
1cg2,4,GLU,A,200,S,LIT,1cg2
1cg2,4,HIS,A,112,S,LIT,1cg2
1cg2,4,ASP,A,141,S,LIT,1cg2

1cg2,4,HIS,A,385,S,LIT,1cg2
1cg2,4,GLU,A,176,S,LIT,1cg2
1i1i,0,TYR,P,613,S,LIT,1i1i
1i1i,0,GLU,P,503,S,LIT,1i1i
1i1i,1,TYR,P,611,S,PSIBLAST,1o8aA
1i1i,1,GLU,P,475,S,PSIBLAST,1o8aA
1i1i,1,ALA,P,426,O,PSIBLAST,1o8aA
1i1i,1,HIS,P,425,S,PSIBLAST,1o8aA
1b12,0,SER,A,90,SN,PSIBLAST,1t7dA
1b12,0,LYS,A,145,S,PSIBLAST,1t7dA
1b12,0,SER,A,88,S,PSIBLAST,1t7dA
1a1r,0,ASP,A,107,S,PSIBLAST,1rgqA
1a1r,0,HIS,A,83,S,PSIBLAST,1rgqA
1a1r,0,SER,A,165,SN,PSIBLAST,1rgqA
1a1r,0,GLY,A,163,N,PSIBLAST,1rgqA
1bef,0,SER,A,135,SN,PSIBLAST,1df9A
1bef,0,GLY,A,153,N,PSIBLAST,1df9A
1e5t,0,ASP,A,641,S,PSIBLAST,1pfqA
1e5t,0,SER,A,554,S,PSIBLAST,1pfqA
1e5t,0,HIS,A,680,S,PSIBLAST,1pfqA
1kzh,0,THR,A,204,S,LIT,1kzh
1kzh,0,ASP,A,206,S,LIT,1kzh
1kzh,0,GLY,A,82,N,LIT,1kzh
1kzh,0,LYS,A,203,S,LIT,1kzh
1kzh,0,ARG,A,146,S,LIT,1kzh
1bx4,0,ASP,A,300,S,PSIBLAST,1lioA
1bx4,1,ASP,A,300,S,PSIBLAST,1rk2A
1bx4,1,ALA,A,298,N,PSIBLAST,1rk2A
1bx4,1,GLY,A,297,N,PSIBLAST,1rk2A
1bx4,1,GLY,A,299,N,PSIBLAST,1rk2A
1b0z,0,GLU,A,285,X,PSIBLAST,1dqrA
1b0z,1,HIS,A,306,X,PSIBLAST,1dqrB
13pk,0,GLY,A,376,N,LIT,13pk
13pk,0,ARG,A,39,S,LIT,13pk
13pk,0,LYS,A,219,S,LIT,13pk
13pk,0,GLY,A,399,N,LIT,13pk
1jxh,0,GLY,A,210,N,LIT,1jxh
1jxh,0,LYS,A,176,S,LIT,1jxh
1a3w,0,GLU,A,334,S,PSIBLAST,1pknA
1a3w,0,THR,A,298,S,PSIBLAST,1pknA
1a3w,0,ARG,A,49,S,PSIBLAST,1pknA
1a3w,0,ARG,A,91,S,PSIBLAST,1pknA
1a3w,0,SER,A,332,S,PSIBLAST,1pknA
1a3w,0,LYS,A,240,S,PSIBLAST,1pknA
1ff3,0,CAS,A,51,S,LIT,1ff3
1ff3,1,CAS,A,51,S,LIT,1ff3
1ff3,0,CYS,A,198,S,LIT,1ff3
1ff3,1,CYS,A,198,S,LIT,1ff3
1ff3,0,GLU,A,94,S,LIT,1ff3
1ff3,1,GLU,A,94,S,LIT,1ff3
1a69,0,ASP,A,204,S,LIT,1a69

1a69,3,ASP,A,204,S,PSIBLAST,1cg6A
1a69,0,ARG,A,217,S,LIT,1a69
1a69,3,ILE,A,206,N,PSIBLAST,1cg6A
1b0p,2,THR,A,31,S,PSIBLAST,2pdaA
1b0p,2,GLU,A,64,S,PSIBLAST,2pdaA
1b0p,2,ARG,A,114,S,PSIBLAST,2pdaA
1dl3,0,ASP,A,126,S,PSIBLAST,1nsjA
1dl3,0,CYS,A,7,S,PSIBLAST,1nsjA
1f75,0,ARG,A,42,S,LIT,1f75
1f75,0,ARG,A,33,S,LIT,1f75
1f75,0,ARG,A,197,S,LIT,1f75
1f75,0,ARG,A,203,S,LIT,1f75
1a3c,0,ARG,A,42,S,PSIBLAST,1l1rA
1a3c,0,ASP,A,83,S,PSIBLAST,1l1rA
1ddz,1,ARG,A,407,S,PSIBLAST,1i6pA
1ddz,1,ASP,A,405,S,PSIBLAST,1i6pA
1fnt,2,GLY,E,80,N,PSIBLAST,1pmaB
1fnt,2,SER,E,174,SN,PSIBLAST,1pmaB
1fnt,2,LYS,E,66,S,PSIBLAST,1pmaB
1ad1,0,ASN,A,11,S,PSIBLAST,1aj0A
1aog,1,PHE,A,199,X,PSIBLAST,1getA
1aog,1,CYS,A,58,X,PSIBLAST,1getA
1aog,1,LYS,A,61,X,PSIBLAST,1getA
1aog,1,CYS,A,53,X,PSIBLAST,1getA
117e,0,GLU,A,117,S,PSIBLAST,1wgiA
1o4u,1,GLU,A,187,S,PSIBLAST,1qprA
1o4u,1,ASP,A,208,S,PSIBLAST,1qprA
1o4u,1,LYS,A,125,S,PSIBLAST,1qprA
1o4u,0,ARG,A,92,S,PSIBLAST,1qprA
1ewx,0,CYS,A,40,S,PSIBLAST,1cqgA
1ewx,0,CYS,A,43,S,PSIBLAST,1cqgA
1pem,0,CYS,A,415,S,PSIBLAST,3r1rA
1pem,0,ASN,A,386,S,PSIBLAST,3r1rA
1pem,0,GLU,A,390,S,PSIBLAST,3r1rA
1pem,0,CYS,A,178,S,PSIBLAST,3r1rA
1bk7,0,GLU,A,84,S,PSIBLAST,1bolA
1bk7,0,HIS,A,88,S,PSIBLAST,1bolA
1h1y,0,HIS,A,69,S,PSIBLAST,1dbtA
1h1y,2,HIS,A,69,S,PSIBLAST,1rpxA
1h1y,0,ASP,A,67,S,PSIBLAST,1dbtA
1h1y,2,ASP,A,38,S,PSIBLAST,1rpxA
1h1y,2,ASP,A,178,S,PSIBLAST,1rpxA
1h1y,2,HIS,A,36,S,PSIBLAST,1rpxA
1h1y,4,ARG,A,120,S,PSIBLAST,1g4pA
1bcc,0,HIS,E,161,X,PSIBLAST,1ndoC
1kbz,0,THR,A,104,S,PSIBLAST,1eq2A
1kbz,1,THR,A,104,S,PSIBLAST,1db3A
1kbz,2,THR,A,104,S,PSIBLAST,1ybvA
1kbz,0,LYS,A,132,S,PSIBLAST,1eq2A
1kbz,1,LYS,A,132,S,PSIBLAST,1db3A
1kbz,2,LYS,A,132,S,PSIBLAST,1ybvA

1kbz,0,TYR,A,128,S,PSIBLAST,1eq2A
1kbz,1,TYR,A,128,S,PSIBLAST,1db3A
1kbz,2,TYR,A,128,S,PSIBLAST,1ybvA
1kbz,1,TYR,A,106,S,PSIBLAST,1db3A
1kbz,2,ASN,A,81,S,PSIBLAST,1ybvA
1bqm,0,HIS,A,539,S,PSIBLAST,1rddA
1woq,0,ASP,A,123,S,PSIBLAST,1q18A
1aa1,1,LYS,B,175,X,PSIBLAST,1rblA
1aa1,1,HIS,B,294,X,PSIBLAST,1rblA
1aa1,1,LYS,B,177,X,PSIBLAST,1rblA
1aa1,1,ASP,B,203,X,PSIBLAST,1rblA
1aa1,1,HIS,B,327,X,PSIBLAST,1rblA
1hqc,0,LEU,A,243,S,PSIBLAST,1g8pA
1a30,0,ASP,A,25,S,LIT,1a30
1a30,1,ASP,A,25,S,PSIBLAST,1hivA
1a30,0,THR,A,26,S,LIT,1a30
1fug,0,LYS,A,269,S,LIT,1fug
1fug,0,LYS,A,265,S,LIT,1fug
1fug,0,ASP,A,271,S,LIT,1fug
1fug,1,LYS,A,245,S,LIT,1fug
1fug,1,ARG,A,244,S,LIT,1fug
1fug,1,LYS,A,165,S,LIT,1fug
1fug,1,HIS,A,14,S,LIT,1fug
1e5l,0,ASP,A,126,S,PSIBLAST,1e5qA
1l1d,0,HIS,A,480,S,LIT,1l1d
1l1d,0,ARG,A,493,S,LIT,1l1d
1l1d,0,CYS,A,495,S,LIT,1l1d
1l1d,0,CYS,A,440,S,LIT,1l1d
1l1d,0,ASP,A,484,S,LIT,1l1d
1brm,0,HIS,A,274,S,LIT,1brm
1brm,0,GLN,A,162,S,LIT,1brm
1h3i,0,TYR,A,335,S,LIT,1h3i
1bj4,0,ARG,A,81,X,PSIBLAST,1cs1C
1bj4,1,GLU,A,75,S,PSIBLAST,1dfoA
1bj4,1,THR,A,254,S,PSIBLAST,1dfoA
1bj4,1,LYS,A,257,S,PSIBLAST,1dfoA
1bj4,2,LYS,A,257,S,PSIBLAST,1fc4A
1bj4,2,HIS,A,231,S,PSIBLAST,1fc4A
1azv,0,ARG,A,143,S,PSIBLAST,2jcwA
1azv,0,HIS,A,63,S,PSIBLAST,2jcwA
1azv,2,HIS,A,63,S,PSIBLAST,1esoA
1aqu,0,SER,A,138,S,PSIBLAST,1hy3A
1aqu,0,LYS,A,48,S,PSIBLAST,1hy3A
1a4i,0,LYS,A,56,S,LIT,1a4i
1afw,0,HIS,A,375,S,LIT,1afw
1afw,2,HIS,A,375,S,PSIBLAST,1qflA
1afw,0,CYS,A,403,S,LIT,1afw
1afw,2,CYS,A,403,S,PSIBLAST,1qflA
1afw,0,GLY,A,405,N,LIT,1afw
1afw,0,CYS,A,125,S,LIT,1afw
1aiq,0,HIS,A,207,S,PSIBLAST,1lcb

1aiq,0,GLU,A,58,S,PSIBLAST,1lcb
1aiq,4,GLU,A,58,S,PSIBLAST,1tysA
1aiq,0,ASP,A,169,S,PSIBLAST,1lcb
1aiq,4,ASP,A,169,S,PSIBLAST,1tysA
1aiq,0,SER,A,167,S,PSIBLAST,1lcb
1aiq,0,ASP,A,205,S,PSIBLAST,1lcb
1aiq,2,SER,A,180,S,PSIBLAST,1b02A
1aiq,2,ASN,A,177,S,PSIBLAST,1b02A
1aiq,4,ARG,A,166,S,PSIBLAST,1tysA
1aiq,4,TYR,A,94,S,PSIBLAST,1tysA
1ag1,0,HIS,O,95,S,PSIBLAST,1htiA
1ag1,0,GLU,O,167,S,PSIBLAST,1htiA
1ag1,0,GLY,O,173,N,PSIBLAST,1htiA
1g4e,0,ALA,A,130,S,PSIBLAST,1g4pA
1cy0,0,TYR,A,319,S,PSIBLAST,1eclA
1cy0,0,ASP,A,111,S,PSIBLAST,1eclA
1cy0,0,GLU,A,9,S,PSIBLAST,1eclA
1b0p,2,ASN,A,996,S,PSIBLAST,2pdaA
1f05,0,LYS,A,142,S,PSIBLAST,1onrA
1f05,0,GLU,A,106,S,PSIBLAST,1onrA
1f05,0,ASP,A,27,S,PSIBLAST,1onrA
1evu,0,CYS,A,314,SN,PSIBLAST,1g0dA
1evu,0,ASP,A,396,S,PSIBLAST,1g0dA
1evu,0,HIS,A,373,S,PSIBLAST,1g0dA
1ay0,2,GLU,A,418,X,PSIBLAST,1dtwB
1ay0,4,GLU,A,418,X,PSIBLAST,1ni4B
1ay0,4,HIS,A,481,X,PSIBLAST,1ni4B
1e3u,1,SER,A,67,X,PSIBLAST,1m6kA
1ffy,0,LYS,A,595,S,PSIBLAST,1a8hA
1ffy,0,LYS,A,598,S,PSIBLAST,1a8hA
1ffy,1,LYS,A,598,S,PSIBLAST,1j09A
1d2r,0,LYS,A,192,S,LIT,1d2r
1d2r,0,LYS,A,195,S,LIT,1d2r
1euq,0,GLU,A,34,S,PSIBLAST,1euyA
1euq,0,LYS,A,270,S,PSIBLAST,1euyA
1euq,1,LYS,A,270,S,PSIBLAST,1j09A
1euq,0,ARG,A,260,S,PSIBLAST,1euyA
1li5,0,LYS,A,266,S,PSIBLAST,1a8hA
1li5,0,LYS,A,269,S,PSIBLAST,1a8hA
1li5,2,LYS,A,269,S,PSIBLAST,1j09A
1asy,1,ARG,A,325,S,LIT,1asy
1asy,1,ARG,A,531,S,LIT,1asy
1asy,1,ASP,A,342,S,LIT,1asy
1adj,0,ARG,A,112,S,PSIBLAST,1qf6A
1b70,0,ARG,A,204,S,PSIBLAST,1b7yA
1a50,3,TYR,A,175,S,PSIBLAST,1geqA
1a50,3,GLU,A,49,S,PSIBLAST,1geqA
1a50,3,ASP,A,60,S,PSIBLAST,1geqA
1k8w,0,ASP,A,48,S,PSIBLAST,1ze1A
1a0h,0,ASP,B,419,S,PSIBLAST,1ds2E
1a0h,2,ASP,B,419,S,PSIBLAST,1qrzA

1a0h,4,ASP,B,419,S,PSIBLAST,1a0jA
1a0h,6,ASP,B,419,S,PSIBLAST,2lprA
1a0h,0,SER,B,525,SN,PSIBLAST,1ds2E
1a0h,2,SER,B,525,SN,PSIBLAST,1qrzA
1a0h,4,SER,B,525,S,PSIBLAST,1a0jA
1a0h,0,GLY,B,523,N,PSIBLAST,1ds2E
1a0h,6,GLY,B,523,N,PSIBLAST,2lprA
1a0h,0,HIS,B,363,S,PSIBLAST,1ds2E
1a0h,2,HIS,B,363,S,PSIBLAST,1qrzA
1a0h,4,HIS,B,363,S,PSIBLAST,1a0jA
1a0h,6,HIS,B,363,S,PSIBLAST,2lprA
1a0h,2,GLY,B,526,N,PSIBLAST,1qrzA
1nb8,0,ASN,A,218,S,PSIBLAST,1nbfA
1nb8,0,CYS,A,223,SN,PSIBLAST,1nbfA
1nb8,0,ASP,A,481,S,PSIBLAST,1nbfA
1nb8,0,HIS,A,464,S,PSIBLAST,1nbfA
1dli,0,ASP,A,264,S,LIT,1dli
1dli,0,LYS,A,204,S,LIT,1dli
1dli,0,ASN,A,208,S,LIT,1dli
1dli,0,CYS,A,260,S,LIT,1dli
1dli,0,THR,A,118,S,LIT,1dli
1dli,0,GLU,A,145,S,LIT,1dli
1j93,0,ASP,A,82,S,PSIBLAST,1uroA
1j93,0,TYR,A,159,S,PSIBLAST,1uroA
